# Numerical simulation of coal particle motion characteristics in the envelope region of spiral drum based on discrete element method

**DOI:** 10.1038/s41598-024-84862-7

**Published:** 2025-01-07

**Authors:** Zhen Tian, Shan Gao, Lianwei Ma, He Wang, Yang Ge

**Affiliations:** 1 College of Mechanical and Electrical Engineering, Zhoukou Normal University, Zhoukou, 466000 China; 2 Mechanical Engineering, Liaoning Technical University, Fuxin, 123000 China; 3 CCTEG Taiyuan Research Institute, Taiyuan, 030006 Shanxi China

**Keywords:** Thin coal seam, Spiral drum, Discrete element, Loading process, Numerical simulation, Engineering, Civil engineering

## Abstract

In order to study the movement characteristics of coal particles in the coal loading process of spiral drums, the spiral drum of a certain type of shearer was taken as the research object, and the intrinsic parameters of the materials were calibrated through the determination results of coal sample properties, the relevant parameters of coal particle adhesion were determined, and a discrete element model of spiral drum coal loading was established. The distribution of coal particle movement subsequent to the fracture of the coal wall was derived through simulation. By spatially dividing the envelope region of the spiral drum along the radial and axial directions, the number and velocity distribution of coal particles in different envelope regions were obtained. The study revealed that the number of coal particles in radial regions III and IV was significantly higher than that in regions I and II. Most of the coal particles in regions III and IV moved outward along the drum axis under the action of the spiral blades, while a small portion moved from regions III and IV towards regions I and II. The coal particles in the axial region near the outer side of the coal wall have a strong ability to flow towards the scraper conveyor, and the probability of coal particles being thrown towards the rear of the spiral drum is higher in the region near the end plate. The increase in traction speed has little effect on the velocity of coal particles in all directions within the envelope region, while the increase of drum rotation speed can significantly improve the velocity of coal particles in each region. Through statistical analysis, it was found that the coal loading rate decreases with the increasing of traction speed. As the drum speed increases, the coal loading rate first increases and then decreases. By comparing the results of industrial experiments and numerical simulations underground, the accuracy of the discrete element method used in this paper to analyze the particle motion in the envelope region of the spiral drum has been confirmed. The research results provide reference for the selection of motion parameters of coal shearer and the improving of coal loading efficiency of spiral drums.

## Introduction

The spiral drum, functioning as the operational mechanism within thin coal seam mining machinery^1,2^, facilitates the conveyance of cut coal blocks to the scraper conveyor through the utilization of spiral blades following the extraction and shearing of the coal seam^3,4^. However, due to various factors such as the operational environment and the structural composition of the spiral drum, its loading efficiency is suboptimal^5,6^. Consequently, enhancing the loading efficiency of the spiral drum poses a formidable challenge encountered in the practical application of thin coal seam mining machinery.

To tackle this issue, numerous experts and researchers have undertaken relevant investigations. Xu^7^ conducted an analysis of the impact of spiral pitch and offset distance of coal blocking plates on the loading rate of the spiral drum through numerical simulation. Gospodarczyk^8^ scrutinized the loading dynamics of thin coal seam mining machinery under different cutting orientations utilizing discrete element software such as PFC. GAO^9^ delved into the loading performance of spiral drums featuring varied hub configurations and structures, proposing a spiral drum design with a curved hub structure. Liu^10^ explored the influence of factors such as coal seam dip angle on the loading efficacy of the spiral drum through numerical simulation, subsequently refining the loading model of the spiral drum using the least squares method. Xu^11^ conducted comparative analyses on the loading effects of spiral drums with conventional and drum-type structures through experimental trials, determining that the drum-type structured spiral drum exhibits superior loading performance. Furthermore, based on experimental findings, adjustments were made to optimize the cone angle of the drum-type hub. Zhao^12^ employed a GA-BP neural network model to forecast the loading performance of the spiral drum under varying structural configurations and motion parameters.

Due to the intricate structural characteristics of the spiral drum, intricate mechanical interactions between coal particles and the drum structure. Traditional mathematical models struggle to adequately capture the nuanced motion characteristics of particles following coal wall fragmentation, while laboratory cutting tests face challenges in effectively monitoring particle motion thereafter^13,14^. Utilizing numerical simulation of the cutting process of the spiral drum, the discrete element method can treat the coal wall as composed of coal particles. Tracking coal particles during the loading process can effectively capture their movement characteristics under nonlinear external action^15–18^.

In the relevant research on coal loading performance, the coal loading performance of the entire spiral drum was analyzed, and did not conduct detailed research on the movement of coal particles^19–24^. Studying the motion of coal particles within the envelope region of the spiral drum, especially investigating the motion characteristics of coal particles in smaller region, can provide a clearer understanding of the motion characteristics of coal particles within the spiral drum. Leveraging the motion dynamics of coal particles, this study investigates particle motion within the envelope region of the spiral drum and develops a simulation model to elucidate the loading process of the spiral drum using EDEM(2019) software. Using a specific type of coal mining machine spiral drum as a case study, discrete element numerical simulation reveals the evolving distribution of coal particle velocities and the variation in coal particle quantities across different envelope regions of the spiral drum during the loading process. Furthermore, the impact of changes in traction speed on coal particle velocity variation across diverse envelope regions of the spiral drum is examined. These research findings provide valuable insights for optimizing the structural design of the spiral drum and enhancing its loading performance.

## Establishment of discrete element model for the coal loading process of spiral drum

**Fig. 1:**
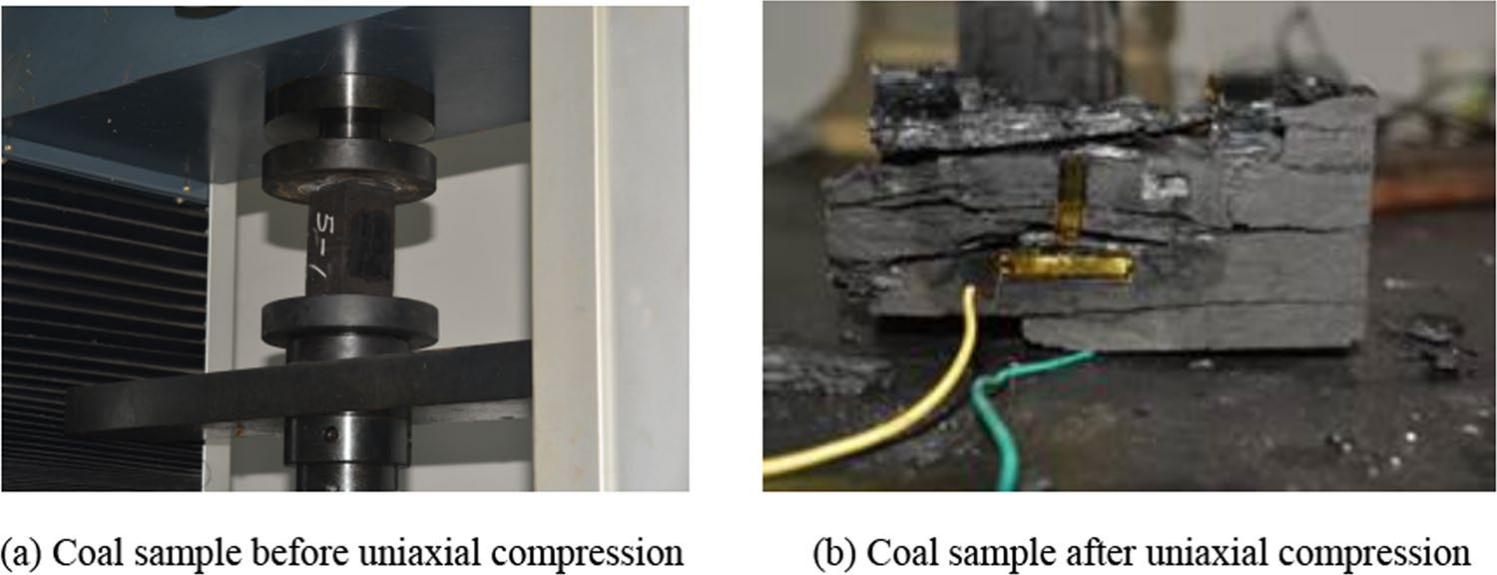
Testing of coal samples.

To enhance the fidelity of the constructed model to real coal walls^25,26^, samples were taken from the coal seams of the Erdos Wenyu coal mine for physical mechanical property testing, as shown in Fig. 1, Mechanical property results of coal samples are shown in Table 1.

**Table 1:** Test results of coal samples.

NO	Tensile strength (MPa)	Compressive strength (MPa)	Elastic modulus (MPa)	Poisson’s ratio	Cohesive force (MPa)	Internal friction angle (°)	Consistent coefficient
5–1	1.08	17.71	4388	0.23	1.85	59	2.0
5–2	0.83	15.78	4112	0.24	1.45	58	1.9

The irregular shapes of coal blocks produced after the fragmentation of actual coal walls present challenges in determining contacts and calculating contact forces due to the complex geometry and collision processes involved^27,28^. To address this, spherical coal particles of equal size were utilized to construct the coal wall model, serving to reflect the motion state of coal particles post fragmentation while facilitating easier detection of contact states among vast particle groups, thereby significantly reducing computational complexity^29,30^.

**Fig. 2:**
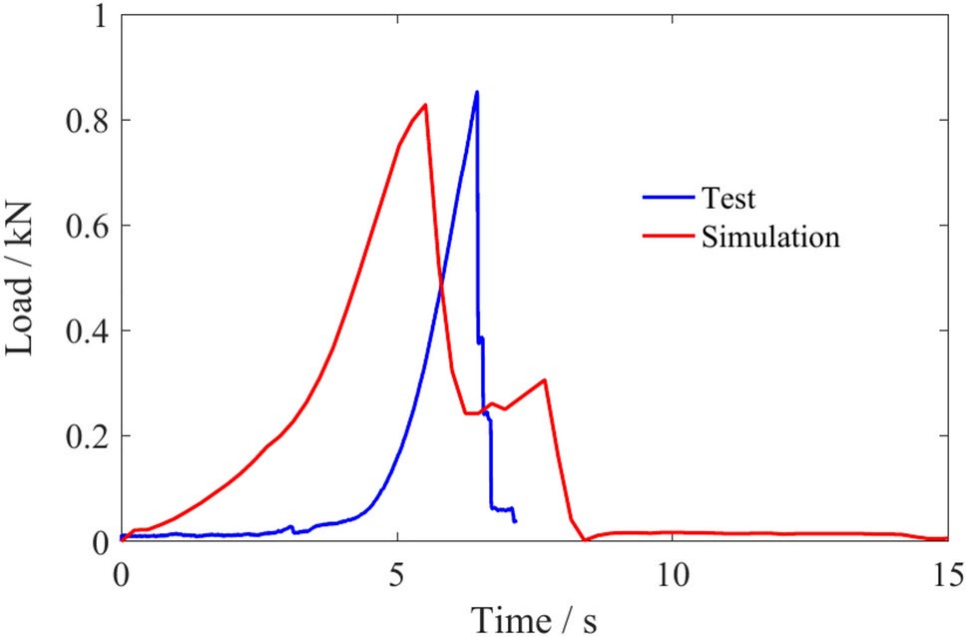
Load of coal samples in two compression tests.

According to the spiral drum cutting principle, the coal particle size should be set less than the distance between sections. The smaller the coal particle size is set, the more it can reflect the movement laws of coal flow. However, due to the large number of small particles required to form the coal wall per unit volume, the number of contacts and collisions between particles increases during the cutting process, which increases the amount of computer calculations and affects the simulation efficiency^31–33^. Combined with the cutting test in reference^5^ and the structure of the spiral drum, the particle radius of the coal was set to 20 mm. Accordingly, based on the results of coal sample property testing, material intrinsic parameters were calculated using the Mohr–Coulomb model^34–36^ and virtual compression test, the bonding parameters for coal particles were set. The load of coal samples in the virtual compression test and actual compression test is shown in Fig. 2, which shows that the parameters in the discrete element model were set accurately.

**Fig. 3:**
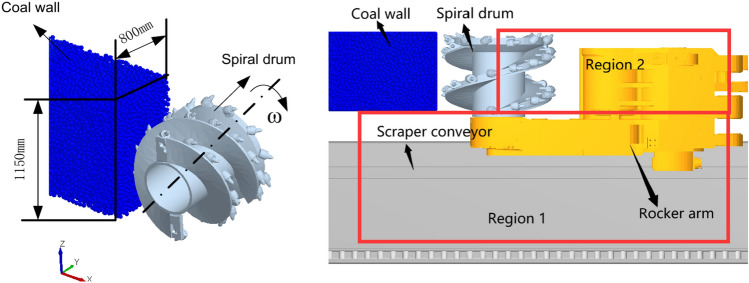
Discrete element model of coal loading process.

These parameters include a bonding radius of 24 mm, normal and tangential stiffness of 21.84 N/m and 12.67 N/m respectively, bonding key normal and shear stresses of 17.71 MPa and 8.16 MPa respectively, static friction coefficient of 0.8 and dynamic friction coefficient of 0.5 between coal particles, and static friction coefficient of 0.7 and dynamic friction coefficient of 0.3 between coal particles and the drum. Based on these parameters, a three-dimensional bonded contact model of the coal wall was constructed^37,38^. Subsequently, following the completion of the coal wall construction, a three-dimensional geometric model of the spiral drum was established, with its material properties set accordingly^39^. The density of coal particles is 1309 kg/m^3^, the drum material was designated as steel, with a density of 7.85 × 10^3^ kg/m^3^, elastic modulus of 4.25 × 10^3^ MPa, and Poisson’s ratio of 0.235. For the purpose of statistical analysis of coal loading rates, the region was divided into region1 and region 2, as illustrated in Fig. 3.

## Numerical simulation of coal loading process with spiral drum

**Fig. 4:**
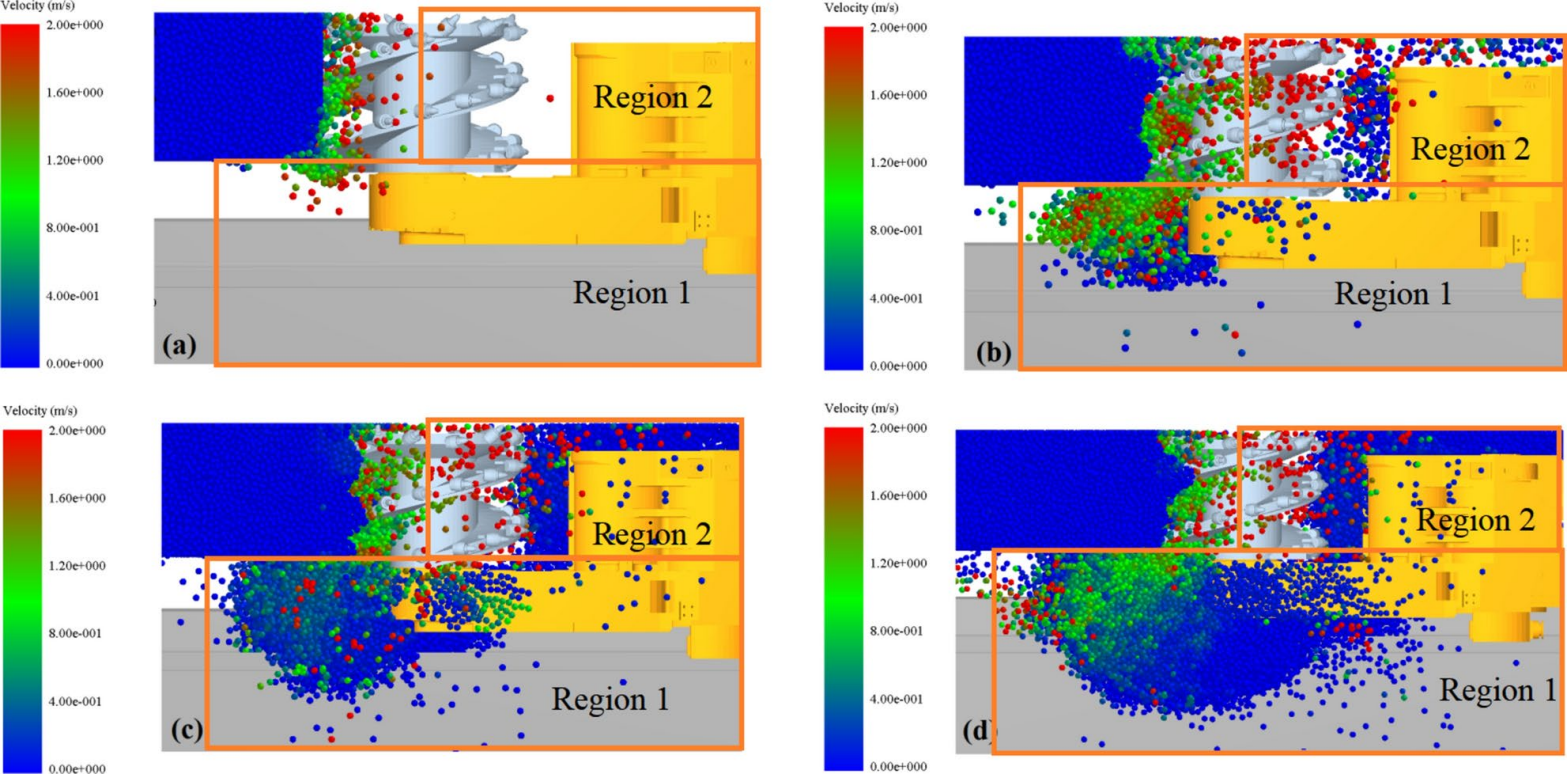
Motion state of coal particles at different times.

A numerical simulation of the coal loading process at a depth of 0.8 m with a traction speed of 4 m/min and a spiral drum rotation speed of 58 r/min was conducted. In the simulation process, we used a PC with a time step set to 20% of the Rayleigh step and a fixed time step of 1.206 × 10^–5^ s. After the simulation is completed, the motion of coal particles can be obtained through EDEM post-processing^39,40^, and the velocity and number of particle changes can be extracted. The resultant velocity process of coal particles during loading shown in Fig. 4. When the spiral drum cuts the coal wall, the coal particles produced by the crushing of the outer coal wall directly enter statistical region 1, while the coal particles produced by the crushing of the inner coal wall enter the envelope region of the spiral drum and flow towards the tail of the spiral drum under the pushing action of the spiral blades. As the cutting continues, the coal particles flowing into both regions continuously increase. The coal particles in region 2, unable to flow into region 1 due to the lack of external forces, remain in the goaf and form floating coal. A portion of coal particles also remains on the rocker arm shell. Meanwhile, most of the coal particles flowing into region 1 gradually accumulate into a circulating coal pile. When the accumulation height reaches a certain level, the coal particles at the top of the pile flow into the conveyor for transportation.

The coal loading rate of the spiral drum can be defined as the ratio of the mass of coal particles in region 1 to the totalmass of coal particles after crushing. The coal loading rate could be obtained:

$$Z = \frac{{Q_{1} }}{{Q_{1} + Q_{2} }} \times 100$$

In the formula: Z is coal loading rate; *Q*_1_ is the mass of coal particles in region 1,in kg; *Q*_2_ is the mass of coal particles in region 2, in kg.

**Fig. 5:**
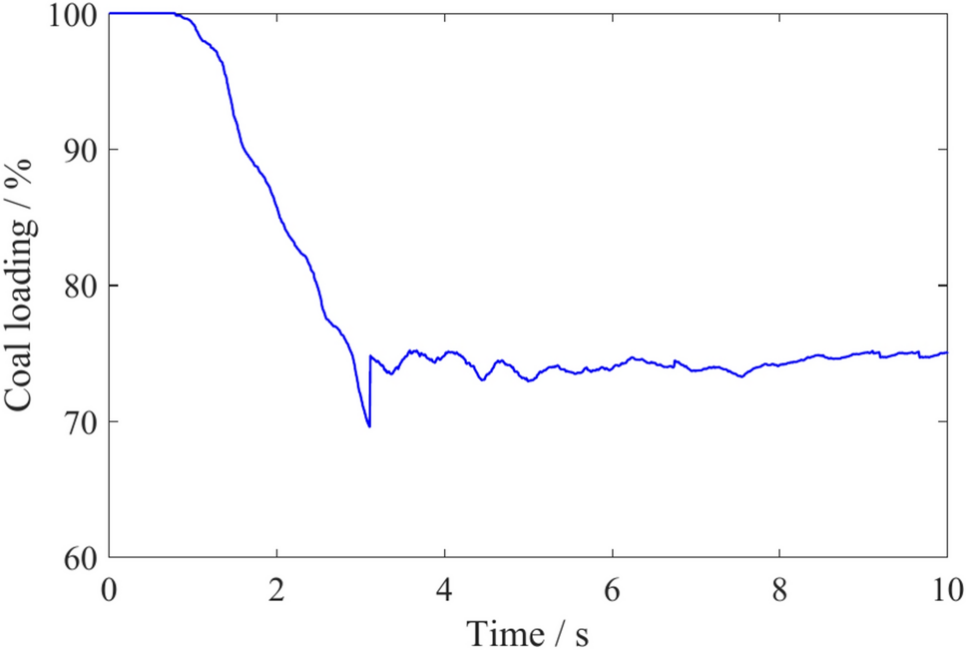
Coal loading rate of spiral drum.

By statistically analyzing simulation data, the coal loading rate of the spiral drum was calculated to be 75.1%, as shown in Fig. 5. Using the coal loading rate theoretical model^30^, the coal loading rate of the spiral drum is calculated to be 68.4%, resulting in a relative error of 8.92% between the simulation results and the theoretical value. This is because during the simulation process, due to the large number of cutting picks installed on the spiral blade, the rotation diameter of the pick tip is larger than the diameter of the spiral blade. At this time, it is equivalent to an increase in the diameter of the spiral blade in numerical simulation, which makes the coal loading rate obtained by numerical simulation higher than the theoretical calculation.

## Analysis of coal particle movement in the spiral drum envelope region

### Analysis of coal particle movement in different regions

**Fig. 6:**
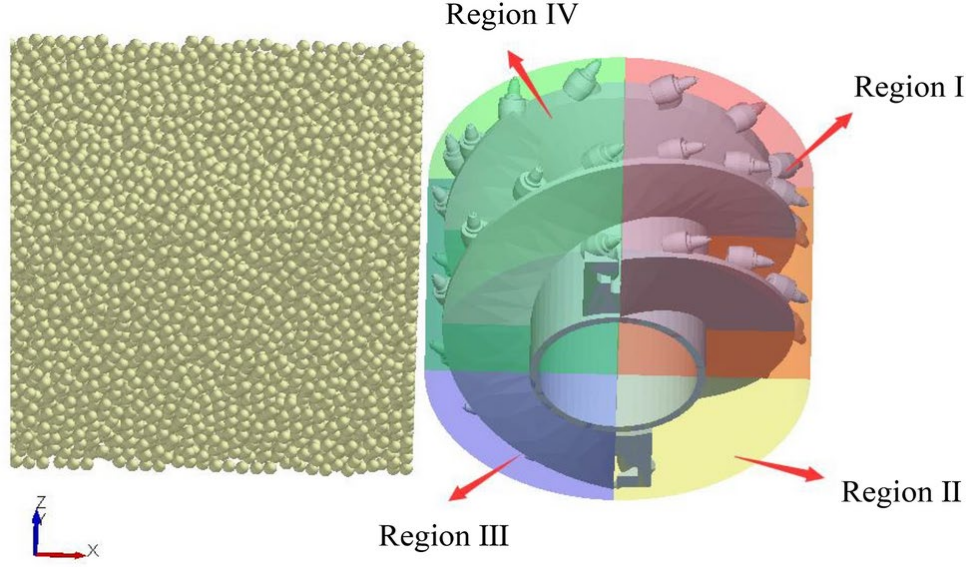
Space division of the drum envelope region.

Due to the complex structure of the spiral drum and the numerous factors affecting the loading process, it is difficult to calculate the motion characteristics of coal particles using mathematical models. By simulating the loading process and treating the coal wall as composed of coal particles using discrete element technology, the motion characteristics of coal particles under nonlinear external forces can be effectively obtained. To analyze the motion characteristics of coal during the loading process, the envelope region of the spiral drum is divided into four spatial regions: I, II, III, and IV, as shown in Fig. 6.

**Fig. 7:**
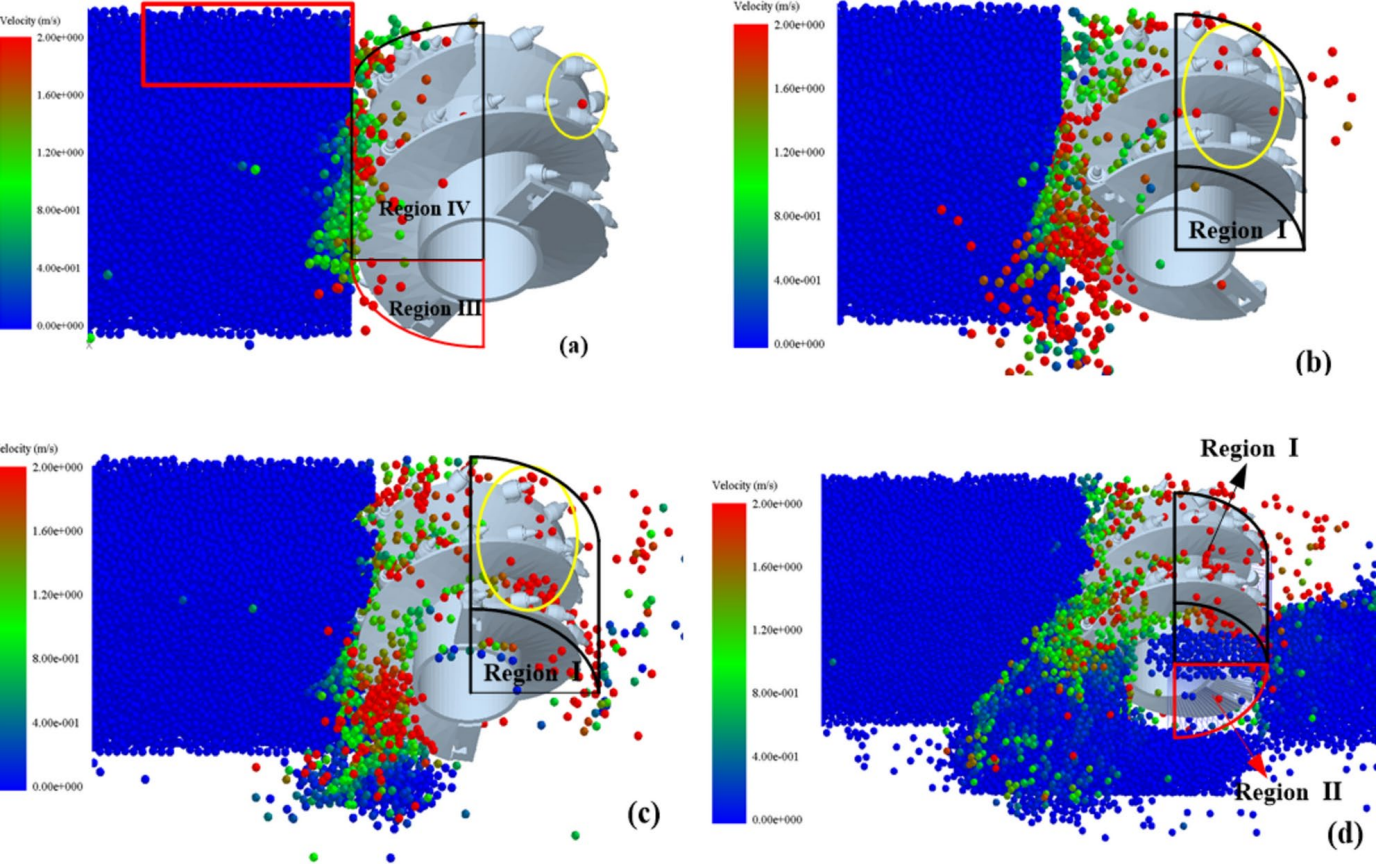
Velocity distribution of coal particles at different times.

Figure 7 shows the velocity distribution of coal particles generated from the crushed coal wall. When the spiral drum enters the coal wall, the coal particles detached from the coal wall enter regions III and IV of the envelope region. Under the pushing action of the spiral blades, the coal particles move in the direction indicated by the arrows in Fig. 7a. When the outward velocity of coal particles in these two regions is less than the upward velocity, some coal particles in both envelope regions cannot flow into transport area and are thrown into mined-out area, as shown by the yellow circles in the figure. As the cutting progresses, the number of coal particles entering regions III and IV increases, leading to a higher filling rate of the envelope region. Due to continuous collision and compression between coal particles, coal particles near the inner wall of the coal wall, close to the end plate position, as indicated by the red box in Fig. 7a, have difficulty flowing into region 1, increasing the probability of being thrown into region 2.

**Fig. 8:**
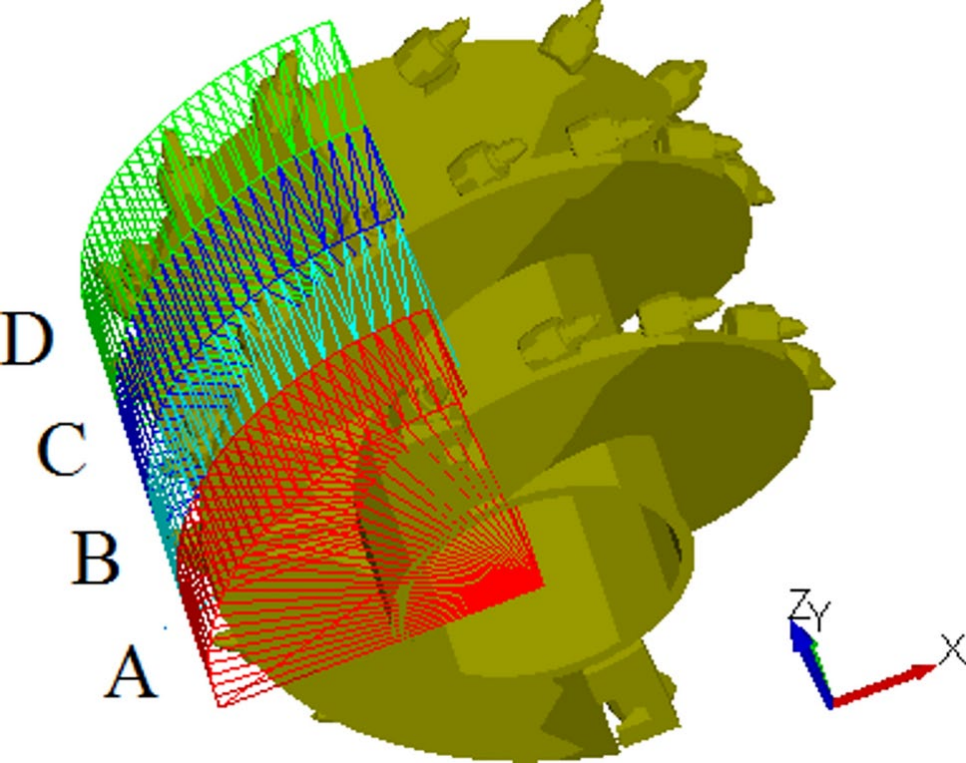
Spatial division of IV region.

To analyze the flow capability of coal particles along the axial direction of the spiral drum during the loading process, region IV was subdivided axially into four small regions: A, B, C, and D, as depicted in Fig. 8.

**Fig. 9:**
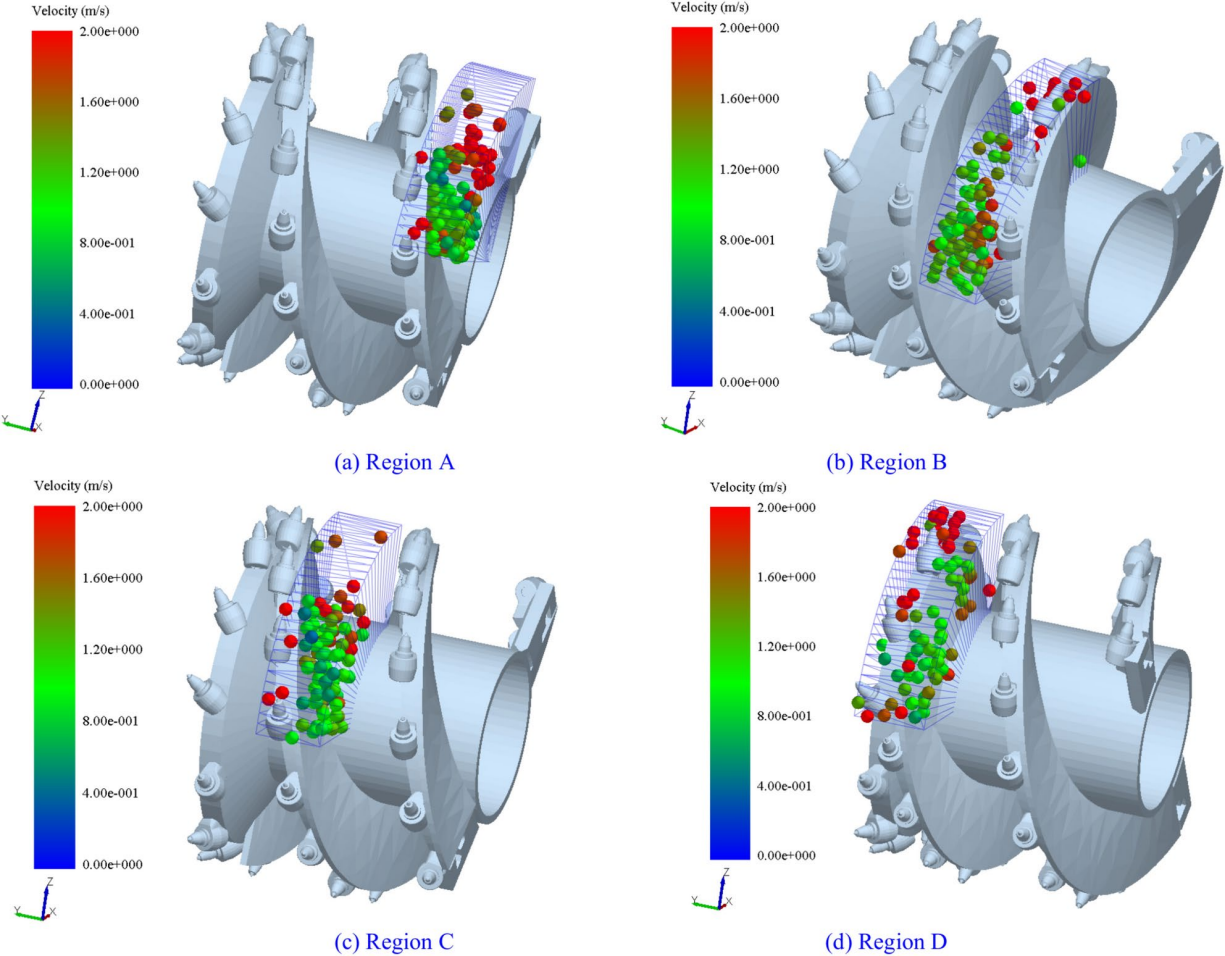
Coal particle velocity distribution in in regions A, B, C, and D.

Figure 9 shows the distribution of coal particles in region IV at different times. As illustrated, with the rotation of the drum, the coal particles in regions A, B, C, and D are constantly changing. Spatial constraints at the intersection of the end disk and the spiral blade result in a lower number of particles in region D relative to the other three regions. In region A, due to its proximity to the tail end of the drum, coal particles from the other three regions continuously flow towards region A under the influence of the spiral blades, leading to a higher number of particles in this region..

**Fig. 10:**
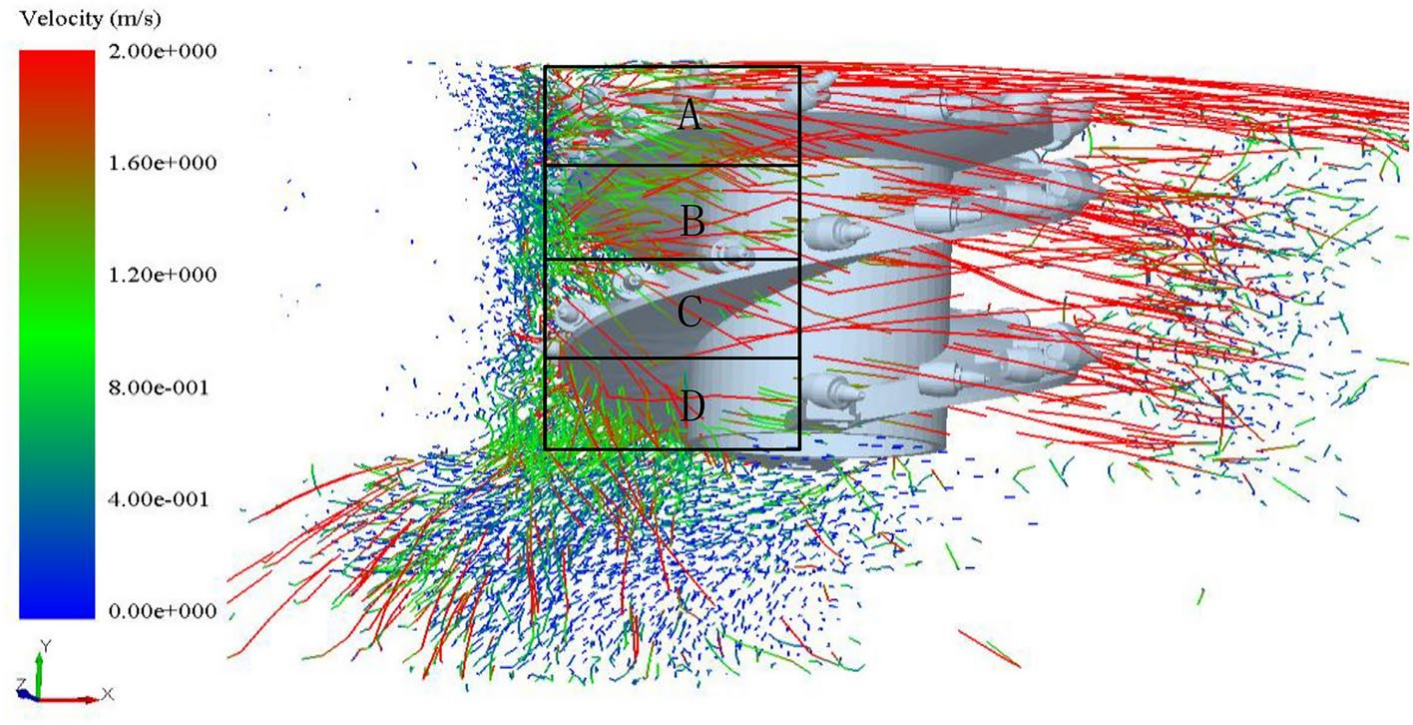
Movement trajectory of coal particles.

Figure 10 shows the motion trajectory of coal particles in the envelope region of the spiral drum during the cutting process. It can be seen from the trajectory diagram that the coal particles in region A mainly move outward along the axial direction of the drum. Most of the coal particles in regions B and C flow towards region A under the push of spiral blades, but some particles were still thrown towards the back of the spiral drum. Due to the absence of spiral blades at the end disk position in region D, most of the coal particles in this region cannot flow outward along the axial direction of the drum.

### The influence of traction speed on coal particle movement

**Fig. 11:**
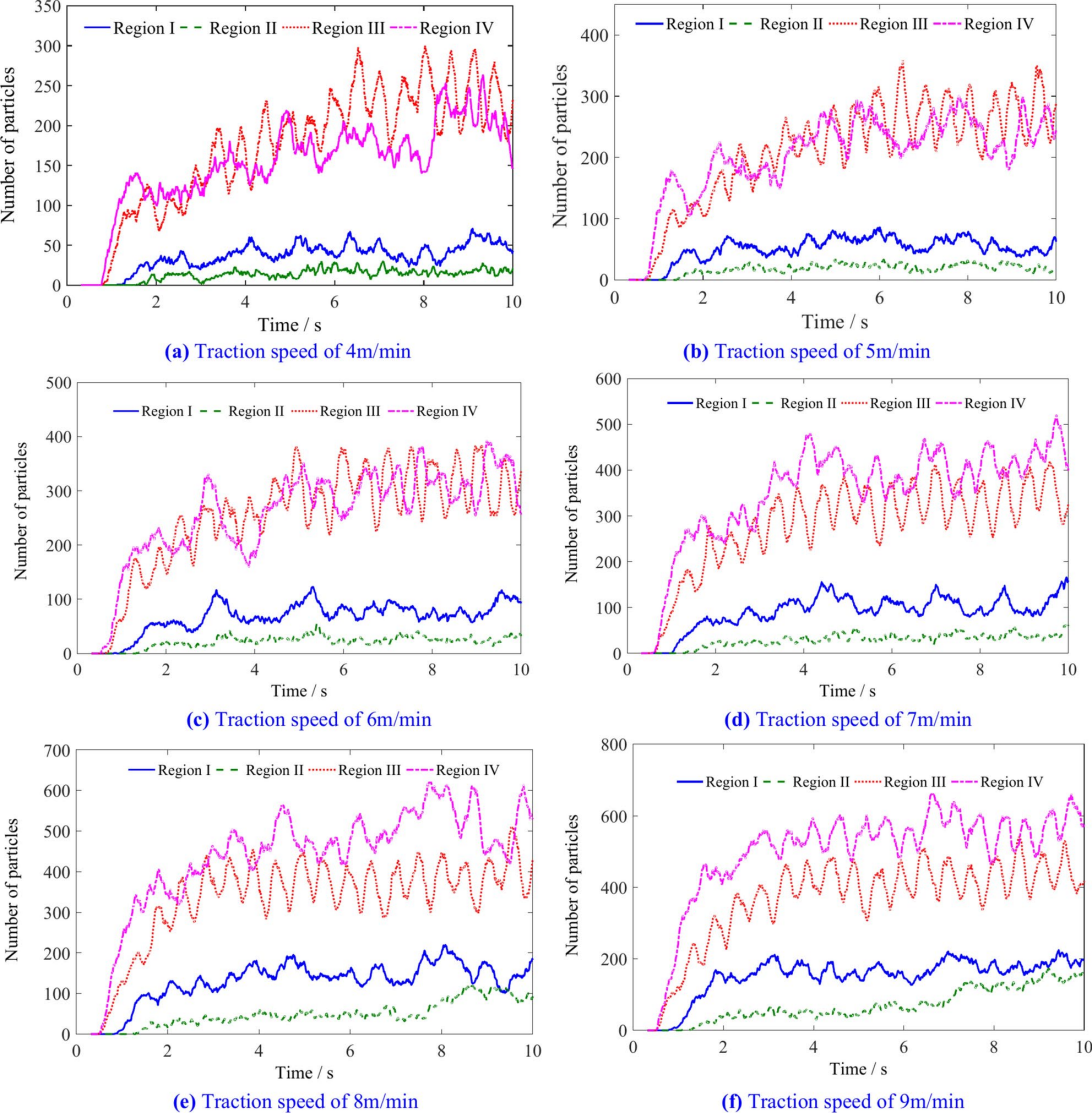
The number of coal particles in regions I, II, III and IV with different traction speeds.

The variation in the number of coal particles in the four envelope regions of the spiral drum with different traction speeds is shown in Fig. 11. After initially contacting and breaking the coal wall, coal particles first enter regions III and IV. With the rotation and advancement of the spiral drum, some coal particles gradually enter region I, and a small portion of them also enter region II under the influence of gravity.

**Fig. 12:**
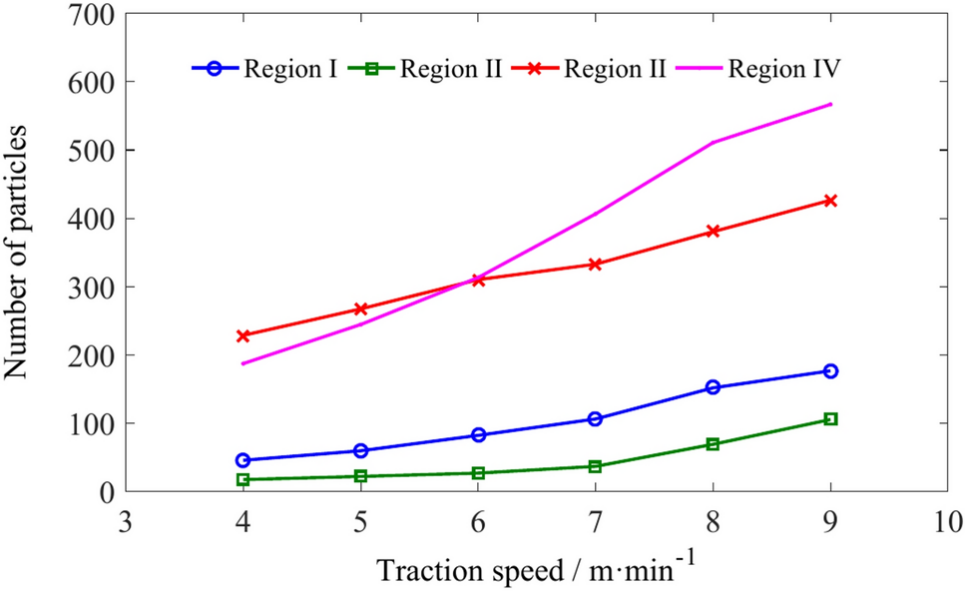
The influence of traction speed on the number of coal particles in regions I, II, III, and IV.

The number of particles of different regions is shown in Table 2. Figure 12 shows the variation pattern of coal particle number in four regions. As the traction speed increases, the number of coal particles in four regions shows an increasing trend. When the traction speed exceeds 6 m/min, the number of particles in region IV exceeds that in region III. Because region III is located below the drum, and as the traction speed increases, the increased number of coal particles in region III increases its filling rate, thereby increasing the probability of coal particles entering region IV under the action of spiral blades.

**Table 2:** The number of coal particles in regions I, II, III and IV.

Traction speed/m min^−1^	Number of coal particles			
	RegionI	Region II	Region III	Region IV
4	45	17	228	187
5	59	22	267	244
6	82	26	310	313
7	106	36	332	406
8	151	69	380	510
9	176	105	426	566

**Fig. 13:**
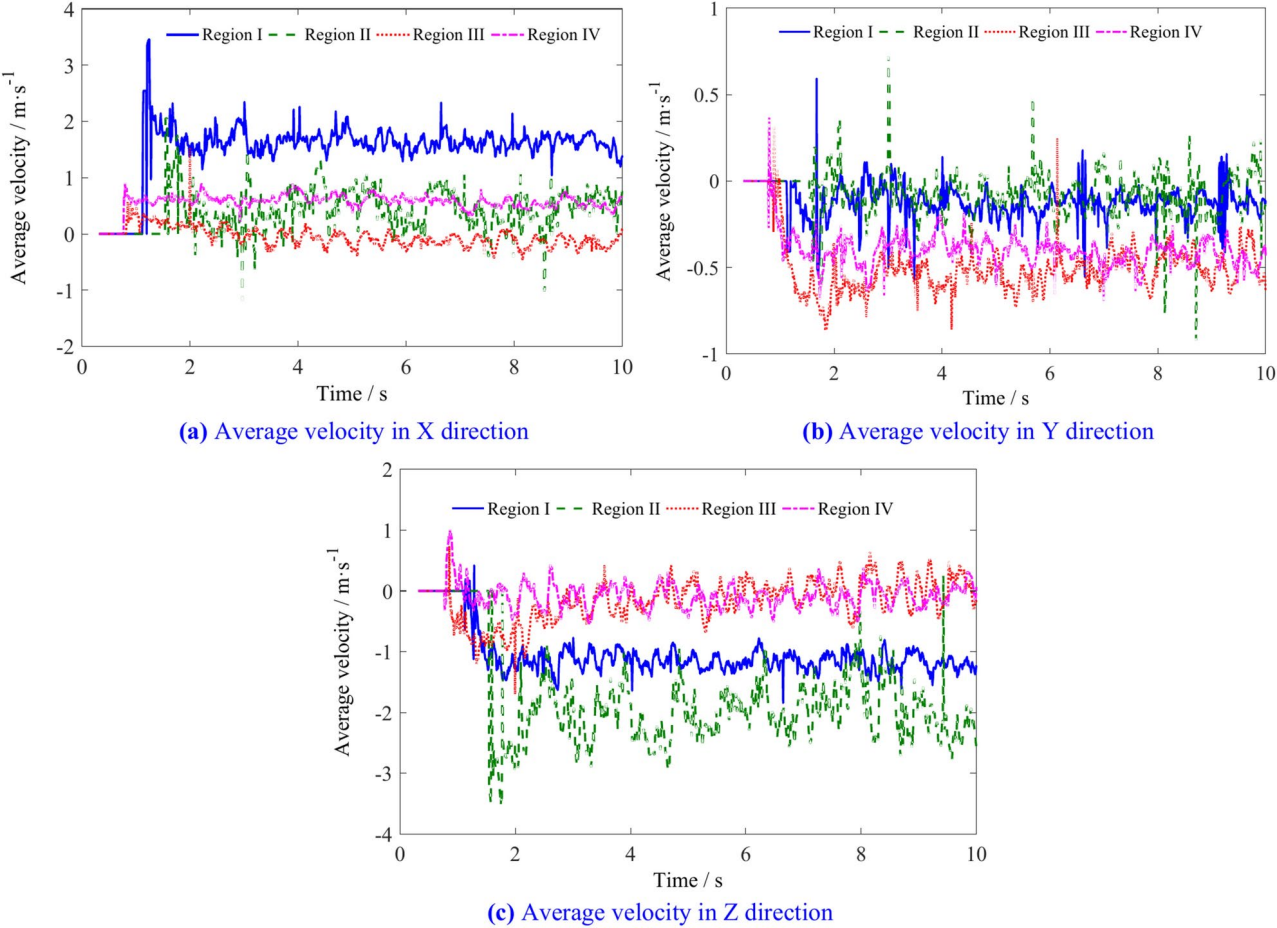
Average velocity of coal particles in regions I, II, III and IV.

The average velocities of the coal particles in the four regions are shown in Fig. 13. The X direction (traction direction) velocity of coal particles in region I is significantly greater than that in the other three regions, indicating a greater probability of coal particles in this region being thrown into the goaf. The X direction velocity of coal particles in region II gradually decreases as the spiral drum continuously cuts into the coal wall. The Y direction (axial direction of drum) velocity of the coal particles in regions III and IV is greater than that in regions I and II, indicating a greater ability of the coal particles in these two regions to flow along the axial direction of the drum. Due to friction from the spiral blades and the increasing number of the coal particles, the Z direction (vertical direction) velocity of the coal particles in region II continuously decreases, while that in regions III and IV is relatively small under the combined action of spiral blades and gravity.

**Fig. 14:**
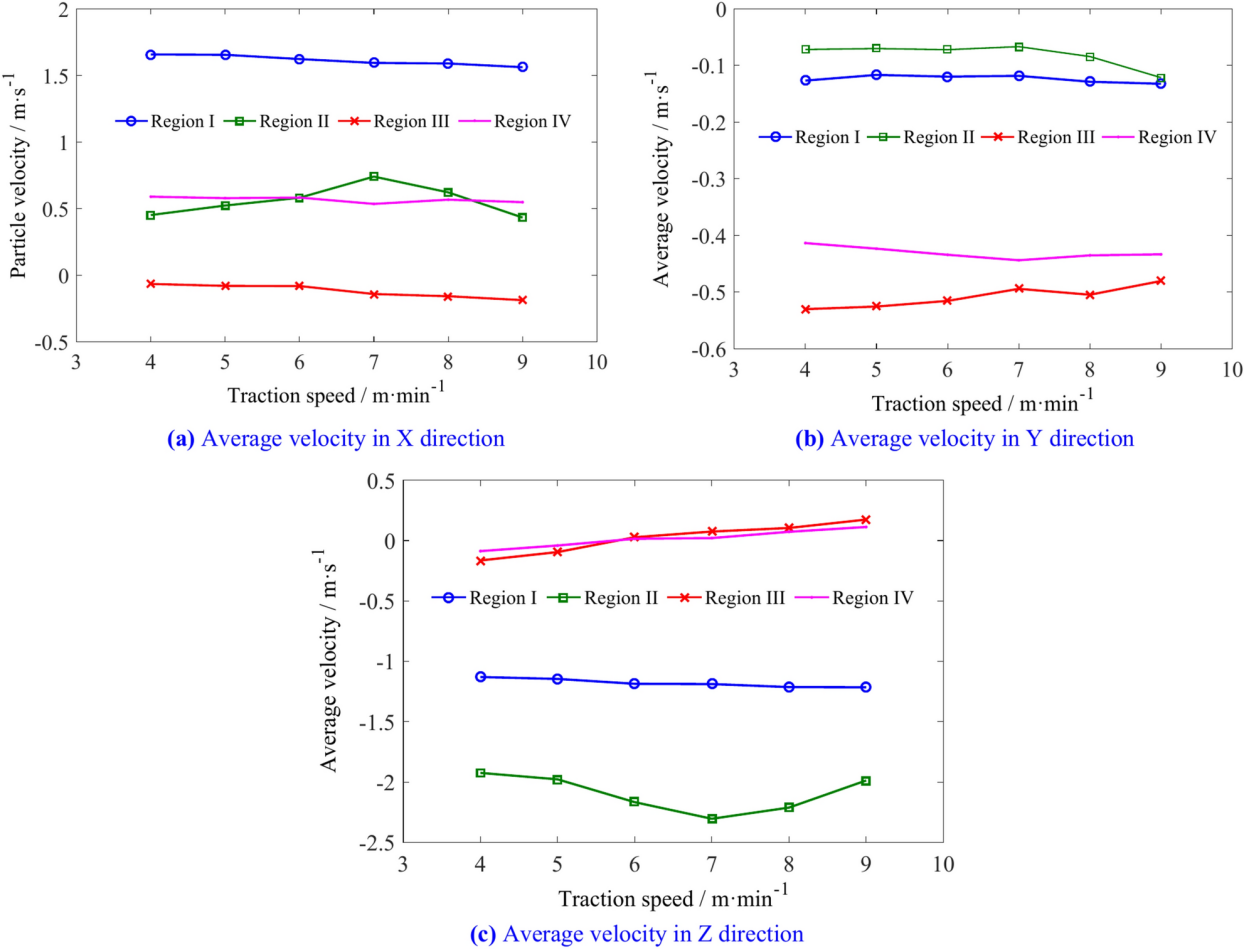
The influence of traction speed on the average velocity of coal particles in regions I, II, III, and IV.

The average velocity of coal particles in regions I, II, III, and IV is shown in Table 3. Figure 14 illustrates the changes in the average velocity of coal particles in different regions with various traction speeds. With increasing traction speeds, the X direction average velocity of coal particles in regions I, III, and IV decreases to some extent, while in region II, the velocity first increases and then decreases. The Y direction velocities of the coal particles in regions I, II, and IV increase, region II showing a larger increase when the speed exceeds 7 m/min, whereas the velocity in region III decreases. In region I, the Z direction velocity increases slightly with increasing traction speed, exhibits an initial increase followed by a decrease in region II, and transitions from negative to positive Y direction in regions III and IV.

**Table 3:** The average velocity of coal particles in regions I, II, III, and IV.

Traction speed/m min^−1^		Average velocity/m s^−1^			
		RegionI	Region II	Region III	Region IV
4	X	1.657	0.451	− 0.066	0.589
	Y	− 0.126	− 0.072	− 0.531	− 0.413
	Z	− 1.131	− 1.925	− 0.165	− 0.087
5	X	1.654	0.522	− 0.081	0.577
	Y	− 0.117	− 0.071	− 0.525	− 0.423
	Z	− 1.146	− 1.9784	− 0.095	− 0.041
6	X	1.623	0.581	− 0.082	0.582
	Y	− 0.119	− 0.072	− 0.515	− 0.4346
	Z	− 1.186	− 2.166	0.027	0.015
7	X	1.5938	0.740	− 0.141	0.534
	Y	− 0.1182	− 0.066	− 0.494	− 0.443
	Z	− 1.188	− 2.304	0.074	0.021
8	X	1.5895	0.621	− 0.158	0.566
	Y	− 0.128	− 0.084	− 0.504	− 0.435
	Z	− 1.213	− 2.211	0.1043	0.072
9	X	1.562	0.432	− 0.1879	0.547
	Y	− 1.215	− 1.987	0.173	0.112
	Z	− 1.215	− 1.987	0.173	0.112

**Fig. 15:**
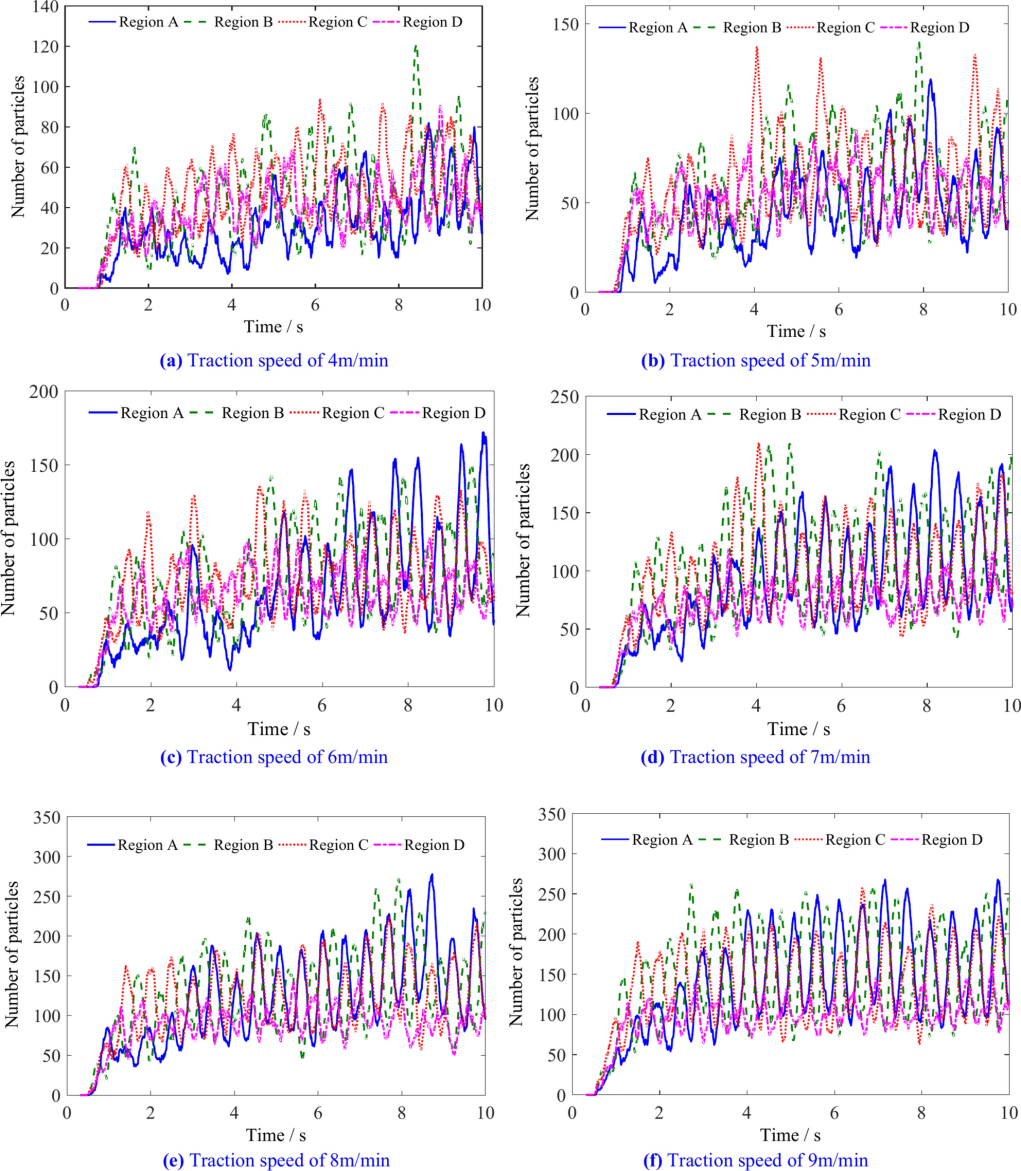
The number of coal particles in A, B, C and D regions with different traction speeds.

The number of particles in the regions A, B, C and D with different traction speeds is shown in Fig. 15. The number of coal particles in different regions shows a periodic change, and gradually increases from the end plate along the axial direction of the drum to the tail of the drum. When the coal particles flowing into region A within a unit time exceed the conveying capacity from that region to the outside, the drum is prone to blockage, which is detrimental to the effective loading of the spiral drum.

**Fig. 16:**
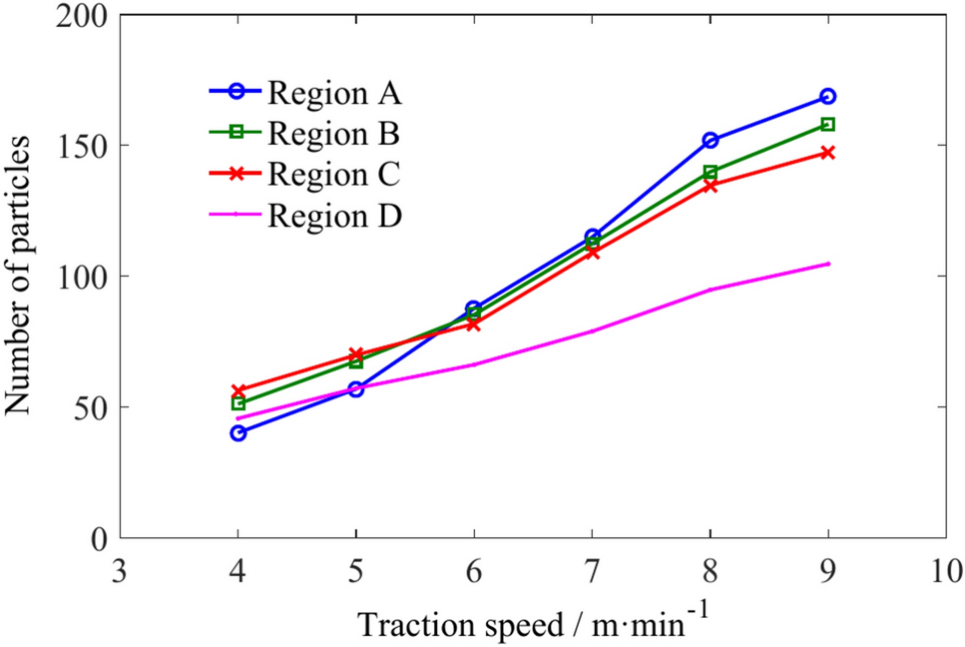
The influence of traction speed on the number of coal particles in regions A, B, C, and D.

The number of coal particles in regions A, B, C, and D is shown in Table 4. Figure 16 shows that the increase in traction speed leads to an increase in the number of particles in regions A, B, C and D. When the traction speed is low, the number of particles in region C is the highest, while the number in region A is the lowest. When the traction speed reaches 6 m/min, the number of particles in regions A, B and C is almost the same. As the traction speed continues to increase, due to the continuous outflow of particles from regions B, C and D, the number of particles in region A eventually reaches its highest value.

**Table 4:** The number of coal particles in regions A, B, C, and D.

Traction speed/m min^−1^	Number of coal particles			
	Region A	Region B	Region C	Region D
4	40	51	56	45
5	56	67	69	57
6	87	85	81	66
7	114	112	108	78
8	151	139	134	94
9	168	158	147	104

**Fig. 17:**
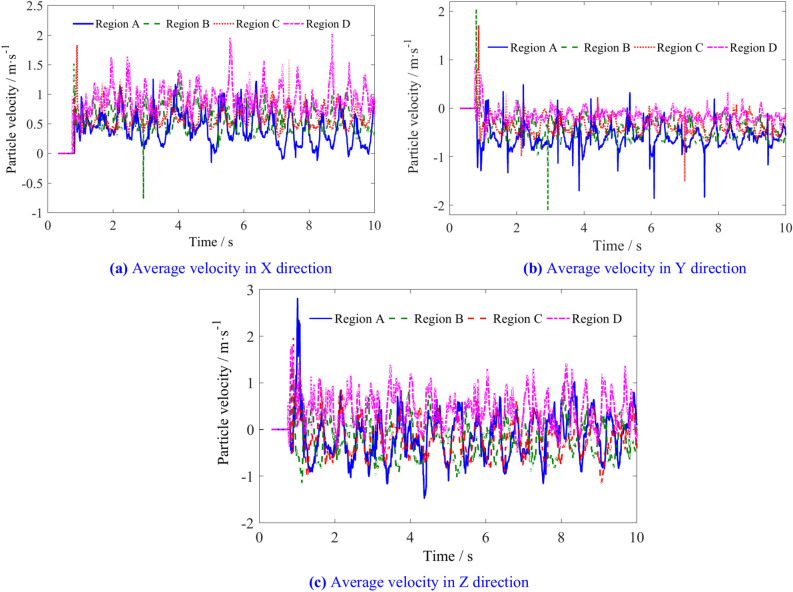
Average velocity of coal particles in regions A, B, C and D.

The average velocities of the coal particles in the four spatial regions are shown in Fig. 17. The X direction velocity of the coal particles in region D is greater than that in the other three regions. Coal particles in this region are more likely to be thrown into the goaf. The X direction velocity of the coal particles in regions A, B and C is proportional to the distance to the tail end of the screw conveyor, while the Y direction velocity in the four regions is inversely proportional to the distance to the tail end of the screw conveyor. The velocity in Z direction in region D is greater than that in the other three regions, and the velocity in the Z direction in regions B and C is lower.

**Fig. 18:**
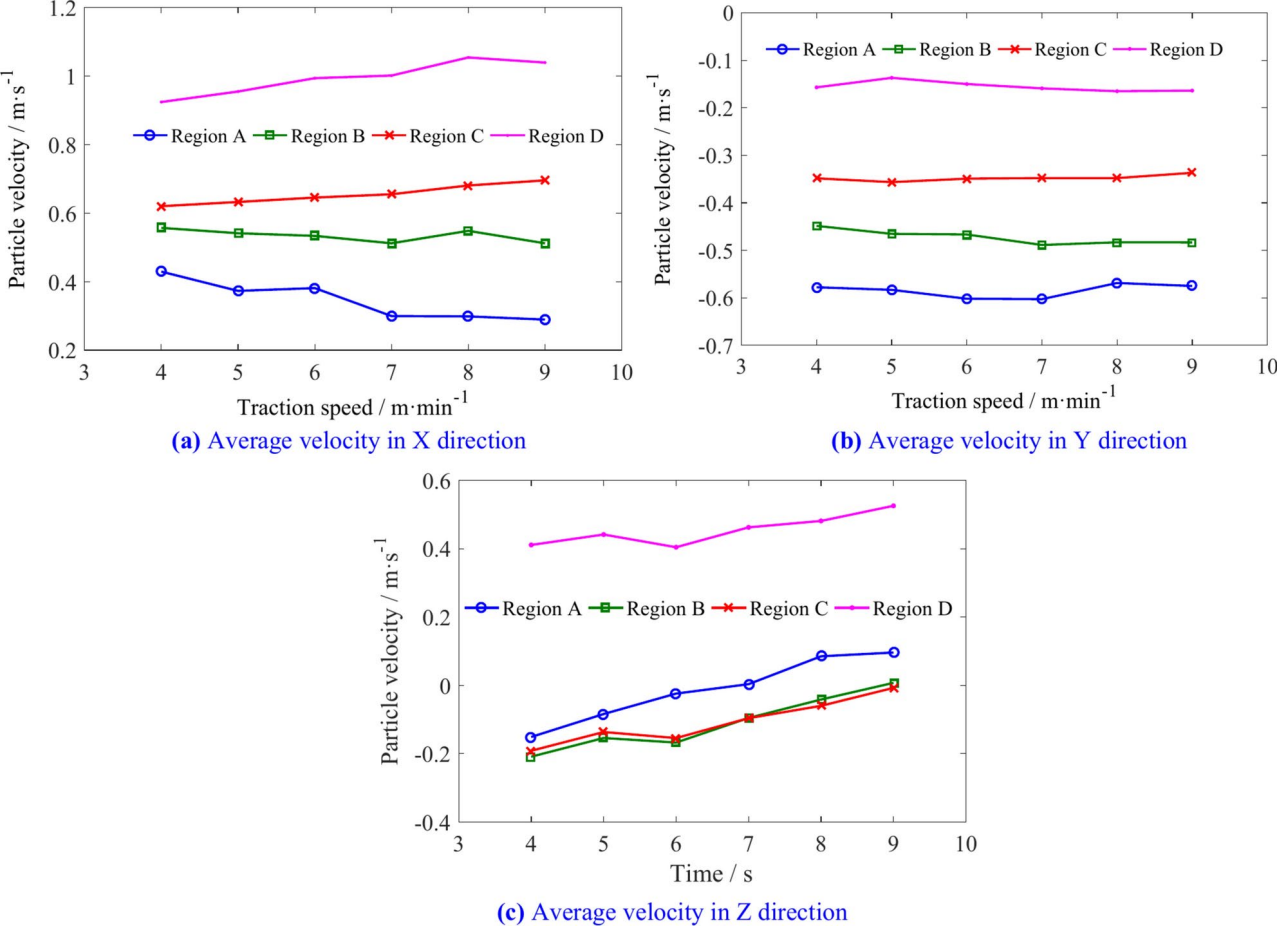
The influence of traction speed on average velocity of coal particles in regions A, B, C, and D.

The average velocities of coal particles in regions A, B, C, and D is shown in Table 5. Figure 18 illustrates the changes in the average velocity of coal particles in different regions with various traction speeds. As the traction speed increases, the velocity of coal particles in region A increases slightly in three directions. The X direction velocity and Z direction velocity of coal particles in region B decrease, while the Y direction velocity increases. In region C, the X direction velocity of coal particles increases, the Y direction velocity remains relatively unchanged, and the Z direction velocity decreases. In region D, the increase in X and Z-direction velocities is greater than in the Y direction. These results indicate that increasing the traction speed can increase the flow capacity of coal particles outward in regions A, B, C, but the probability of coal particles in the region D entering region 2 increases.

**Table 5:** The average velocities of coal particles in regions A, B, C, and D.

Traction speed/m min^−1^		Average velocity/m s^−1^			
		Region A	Region B	Region C	Region D
4	X	0.429	0.557	0.620	0.925
	Y	− 0.578	− 0.449	− 0.348	− 0.157
	Z	− 0.151	− 0.209	− 0.191	0.411
5	X	0.373	0.541	0.633	0.955
	Y	− 0.583	− 0.465	− 0.356	− 0.137
	Z	− 0.084	− 0.154	− 0.137	0.441
6	X	0.381	0.534	0.646	0.994
	Y	− 0.602	− 0.467	− 0.349	− 0.150
	Z	− 0.024	− 0.167	− 0.154	0.404
7	X	− 0.603	− 0.489	− 0.348	− 0.159
	Y	0.300	0.512	0.655	1.002
	Z	0.004	− 0.095	− 0.096	0.462
8	X	0.299	0.548	0.681	1.055
	Y	− 0.569	− 0.483	− 0.348	− 0.165
	Z	0.085	− 0.041	− 0.060	0.481
9	X	0.289	0.512	0.696	1.040
	Y	− 0.575	− 0.483	− 0.337	− 0.164
	Z	0.096	0.007	− 0.007	0.525

### The influence of drum rotation speed on coal particle motion

**Fig. 19:**
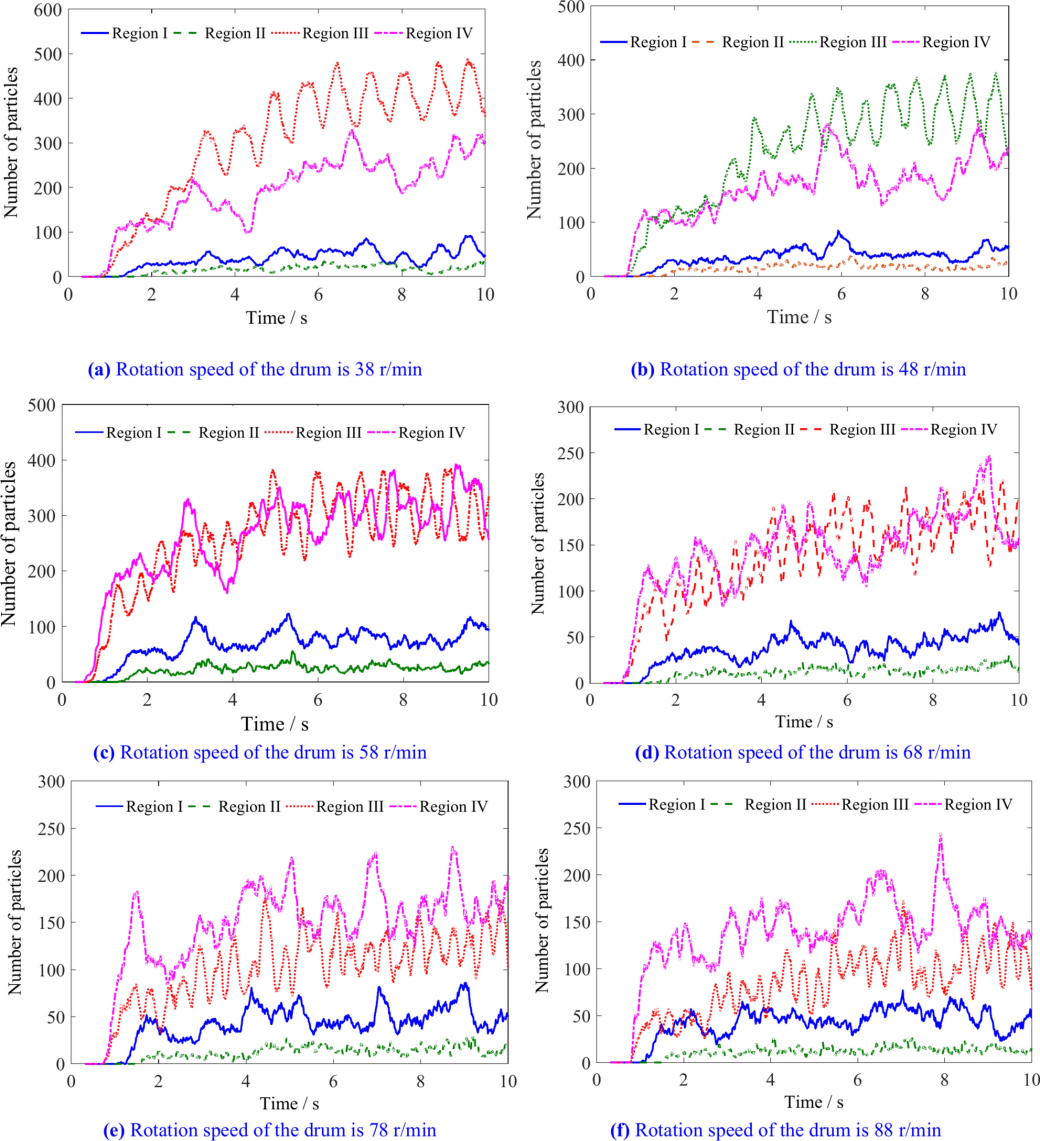
The number of coal particles in regions I, II, III and IV with different rotation speeds.

**Fig. 20:**
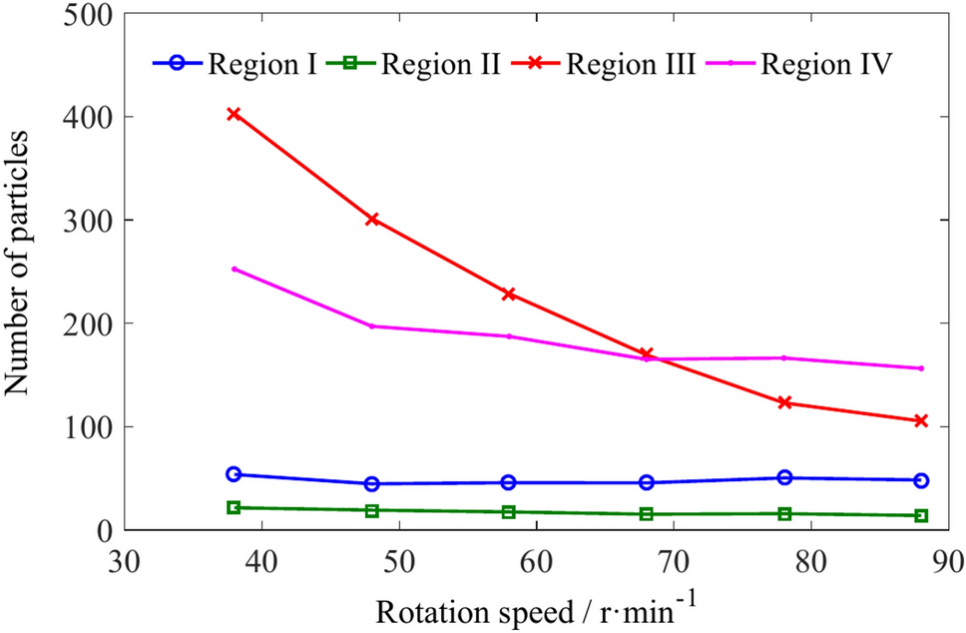
The influence of rotation speed on the number of coal particles in regions I, II, III and IV.

The number and variation pattern of coal particles in regions I, II, III, and IV with different rotational speed conditions are shown in Figs. 19 and 20. The number of coal particles in regions I, II, III and IV is shown in Table 6. The region with the lowest number of coal particles among the four regions is region II, followed by region I. There are more particles in regions III and IV. When the rotation speed is low, there are more coal particles in region III than in region IV. When the rotation speed exceeds 68 r/min, there are more coal particles in region IV than in region III. The increase in rotation speed has no significant effect on the number of coal particles in regions I and II, but as the rotation speed increases, the number of coal particles in regions III and IV continuously decreases, with the decrease in coal particle number in region III being more pronounced.

**Table 6:** The number of coal particles in regions I, II, III and IV.

Rotation speed/r min^−1^	Number of coal particles			
	Region I	Region II	Region III	Region IV
38	54	21	403	252
48	45	19	301	197
58	46	17	229	187
68	46	15	169	165
78	50	16	123	166
88	48	14	105	156

**Fig. 21:**
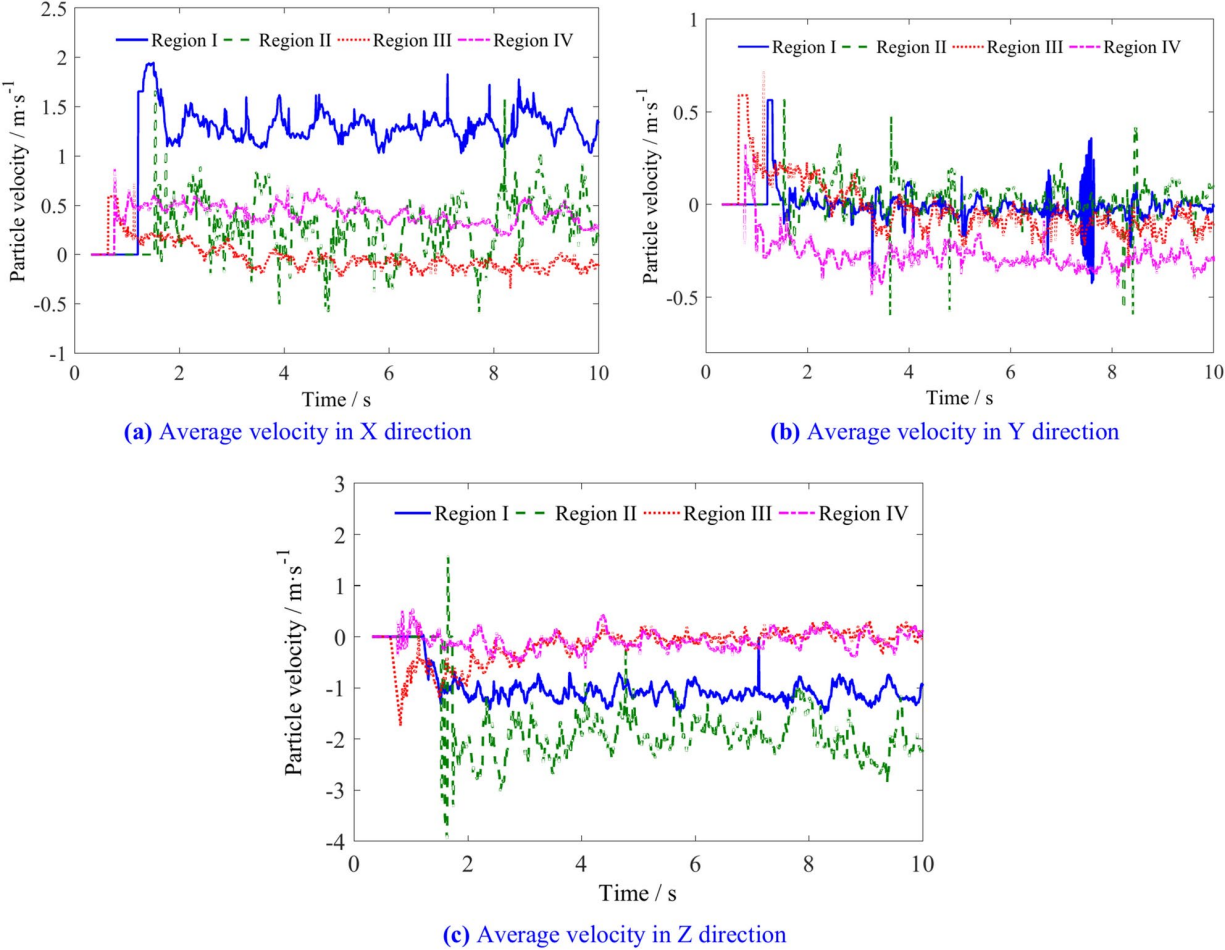
The average velocity of coal particles in regions I, II, III, and IV with a rotation speed of 38 r/min.

When the rotation speed of the drum is 38 r/min, the average velocities of the coal particles in regions I, II, III, and IV is shown in Fig. 21. The X direction velocity of coal particles in region I is significantly greater than that in the other three regions, while the X direction velocity of the coal particles in region III is the smallest. The Y-direction velocity of coal particles in region IV is the highest, while the Z-direction velocity of coal particles in regions I and II is relatively high.

**Fig. 22:**
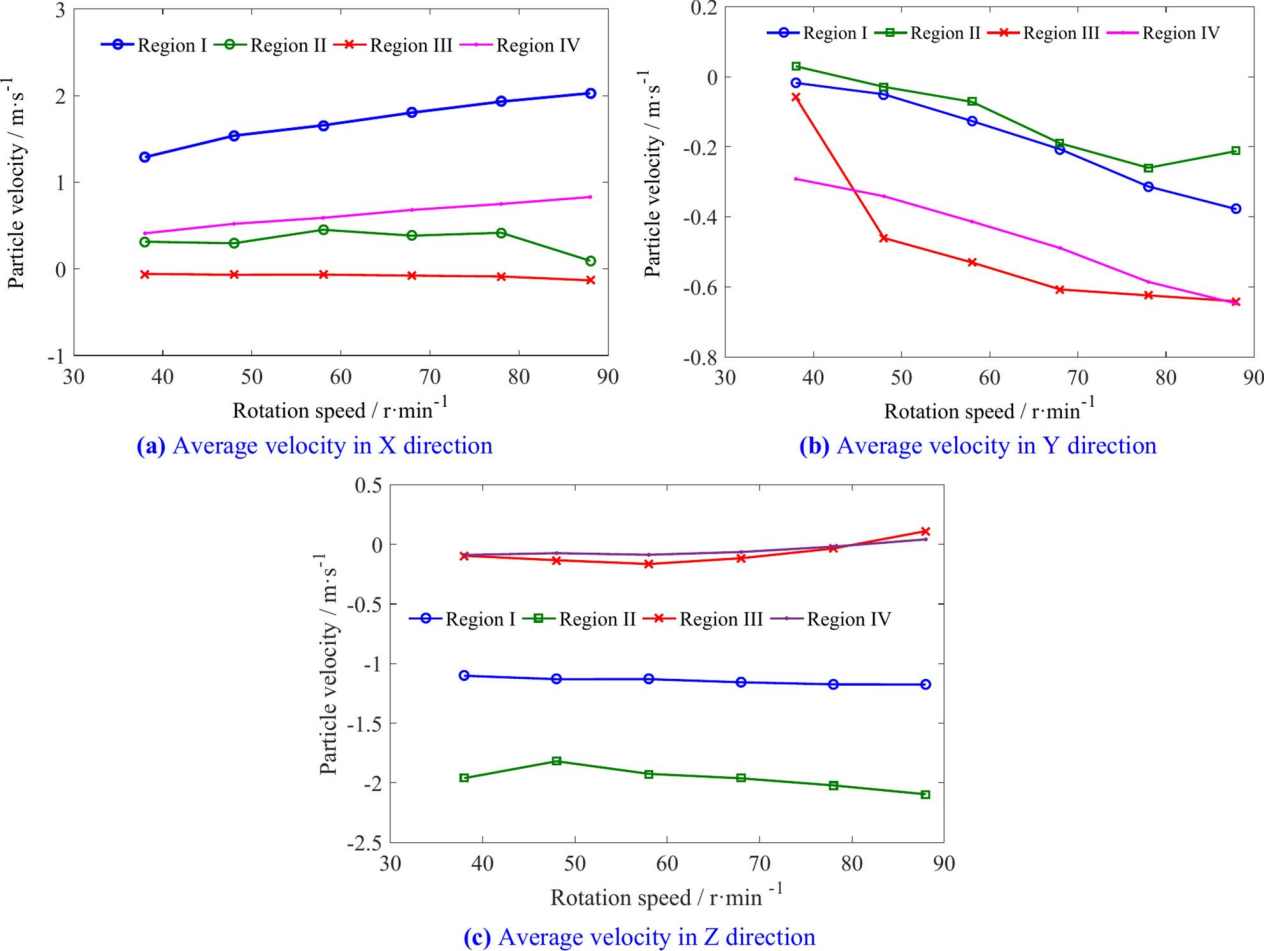
The influence of rotation speed on average velocity of coal particles in regions I, II, III, and IV.

The average velocities of coal particles in regions I, II, III, and IV is shown in Table 7. The average velocity variation of coal particles in different regions is shown in Fig. 22. An increase in the rotation speed causes an increase in the X direction velocity of coal particles in regions I, III, and IV. However, the increase in particle velocity in region III is relatively small, and there is a certain fluctuation in the X direction velocity of coal particles in region II. The velocity of coal particles in the negative Y direction in the four regions increases continuously with the increasing of rotation speed, but shows different patterns of change. The increase in rotation speed has little effect on the Z direction velocity in various regions.

**Table 7:** The average velocities of coal particles in regions I, II, III, and IV.

Rotation speed/r min^−1^		Average velocity/m s^−1^			
		Region I	Region II	Region III	Region IV
4	X	1.290	0.313	− 0.059	0.411
	Y	− 0.018	0.030	− 0.059	− 0.292
	Z	− 1.102	− 1.961	− 0.095	− 0.089
5	X	1.538	0.296	− 0.068	0.520
	Y	− 0.050	− 0.029	− 0.460	− 0.341
	Z	− 1.131	− 1.819	− 0.133	− 0.074
6	X	1.657	0.451	− 0.066	0.589
	Y	− 0.127	− 0.072	− 0.530	− 0.414
	Z	− 1.131	− 1.925	− 0.165	− 0.087
7	X	1.804	0.383	− 0.078	0.681
	Y	− 0.207	− 0.190	− 0.607	− 0.489
	Z	− 1.157	− 1.962	− 0.116	− 0.064
8	X	1.931	0.417	− 0.088	0.749
	Y	− 0.313	− 0.260	− 0.624	− 0.586
	Z	− 1.174	− 2.022	− 0.035	− 0.019
9	X	2.028	0.093	− 0.132	0.829
	Y	− 0.378	− 0.212	− 0.641	− 0.647
	Z	− 1.175	− 2.096	0.111	0.042

**Fig. 23:**
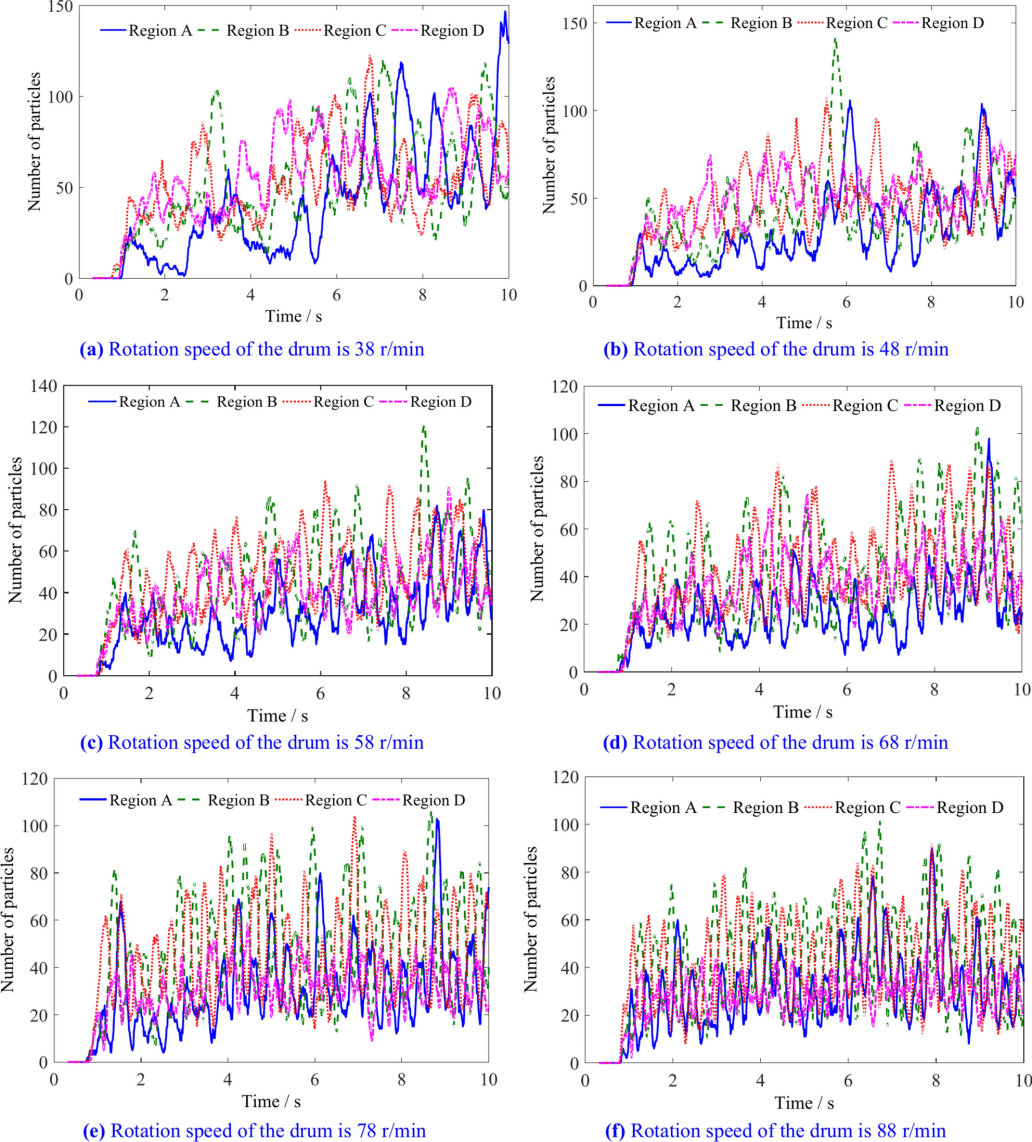
The number of coal particles in regions A, B, C and D with different rotation speeds.

**Fig. 24:**
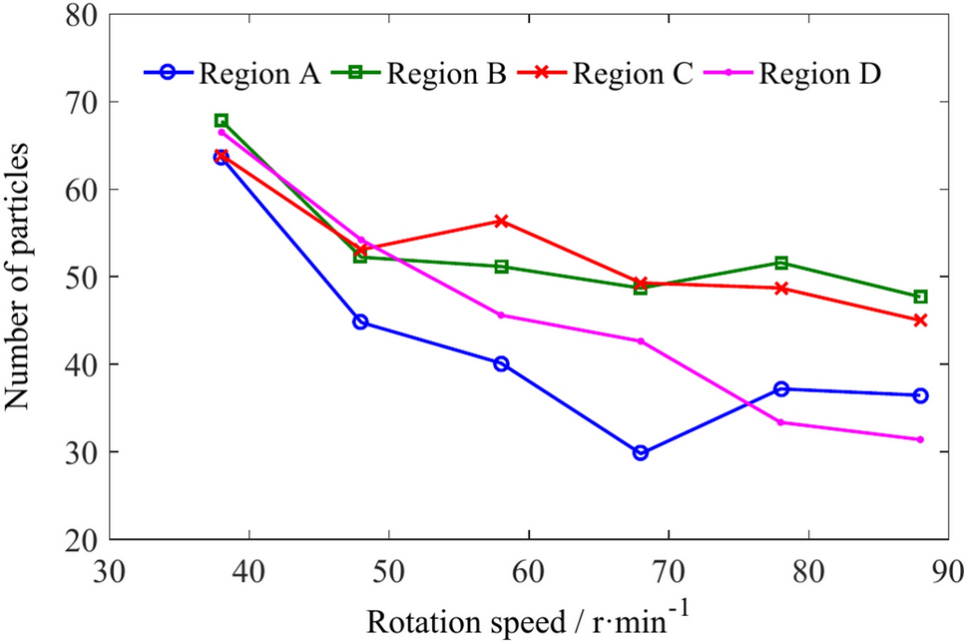
The influence of rotation speed on the number of coal particles in regions A, B, C and D.

The number and variation of coal particles in regions A, B, C, and D of the spiral drum with different rotation speed are shown in Figs. 23 and 24. The Number of particles in regions A, B, C and D is shown in Table 8. When the rotation speed of the spiral drum is low, the number of coal particles in the four regions does not differ significantly. As the rotation speed increases, the ability of coal particles to flow outward along the drum axis in the four regions is enhanced, and the number of coal particles in each region decreases to varying degrees.

**Table 8:** Number of particles in regions A, B, C and D.

Rotation speed/r min^−1^	Number of coal particles			
	Region A	Region B	Region C	Region D
38	64	68	64	66
48	45	52	53	54
58	40	51	56	46
68	30	49	49	43
78	37	52	49	33
88	36	48	45	31

**Fig. 25:**
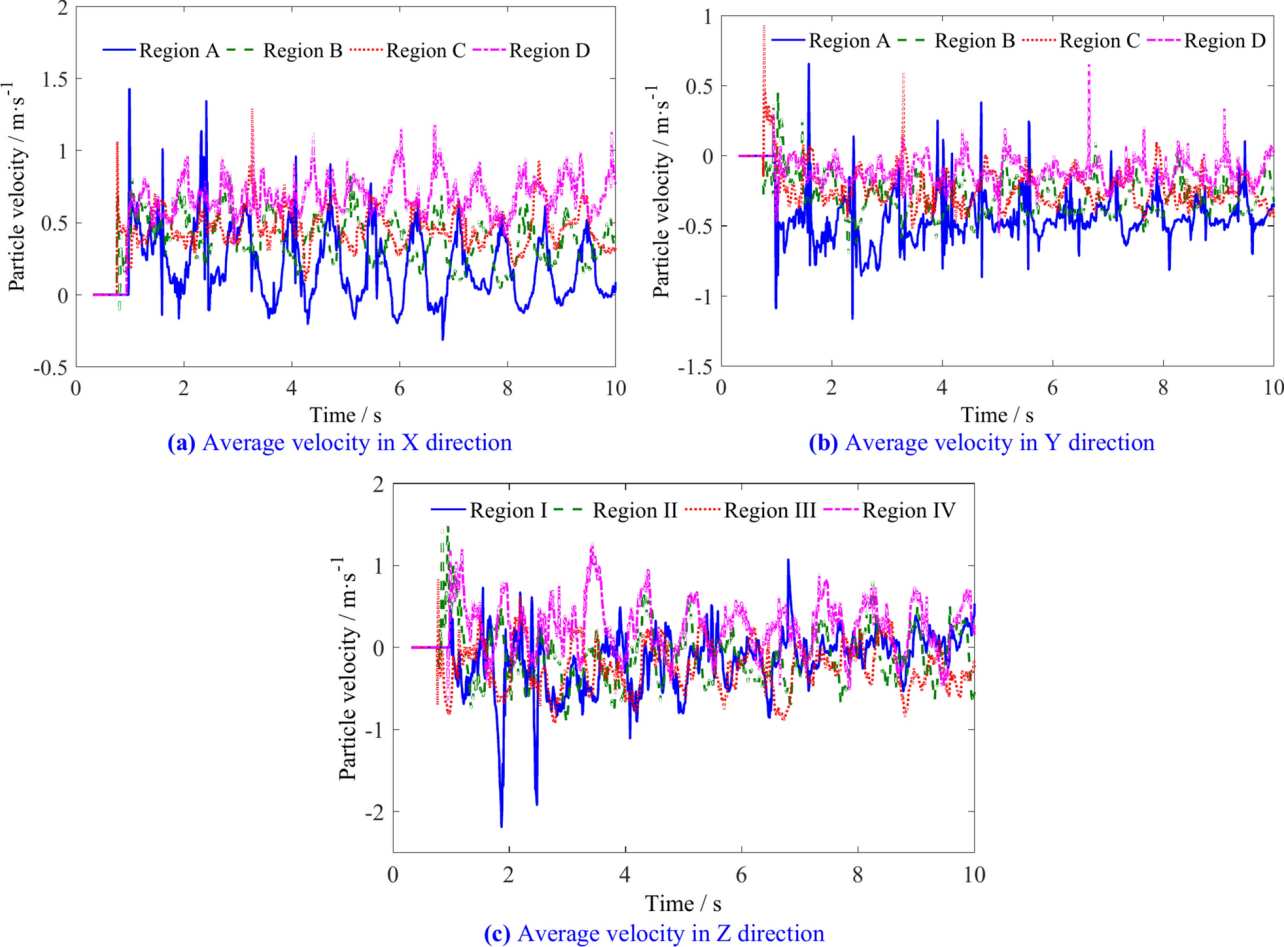
The average velocity of coal particles in regions A, B, C and D with a rotation speed of 38 r/min.

When the rotation speed of the drum is 38 r/min, the average speed of coal particles in regions A, B, C, and D is shown in Fig. 25. The average velocity of coal particles in the four regions in the X direction is *v*_DX_ > *v*_CX_ > *v*_BX_ > *v*_AX_, the average velocity in the Y direction is *v*_AX_ > *v*_BX_ > *v*_CX_ > *v*_DX_, and the average velocity in the Z direction is *v*_DX_ > *v*_CX_ > *v*_AX_ > *v*_BX_.

**Fig. 26:**
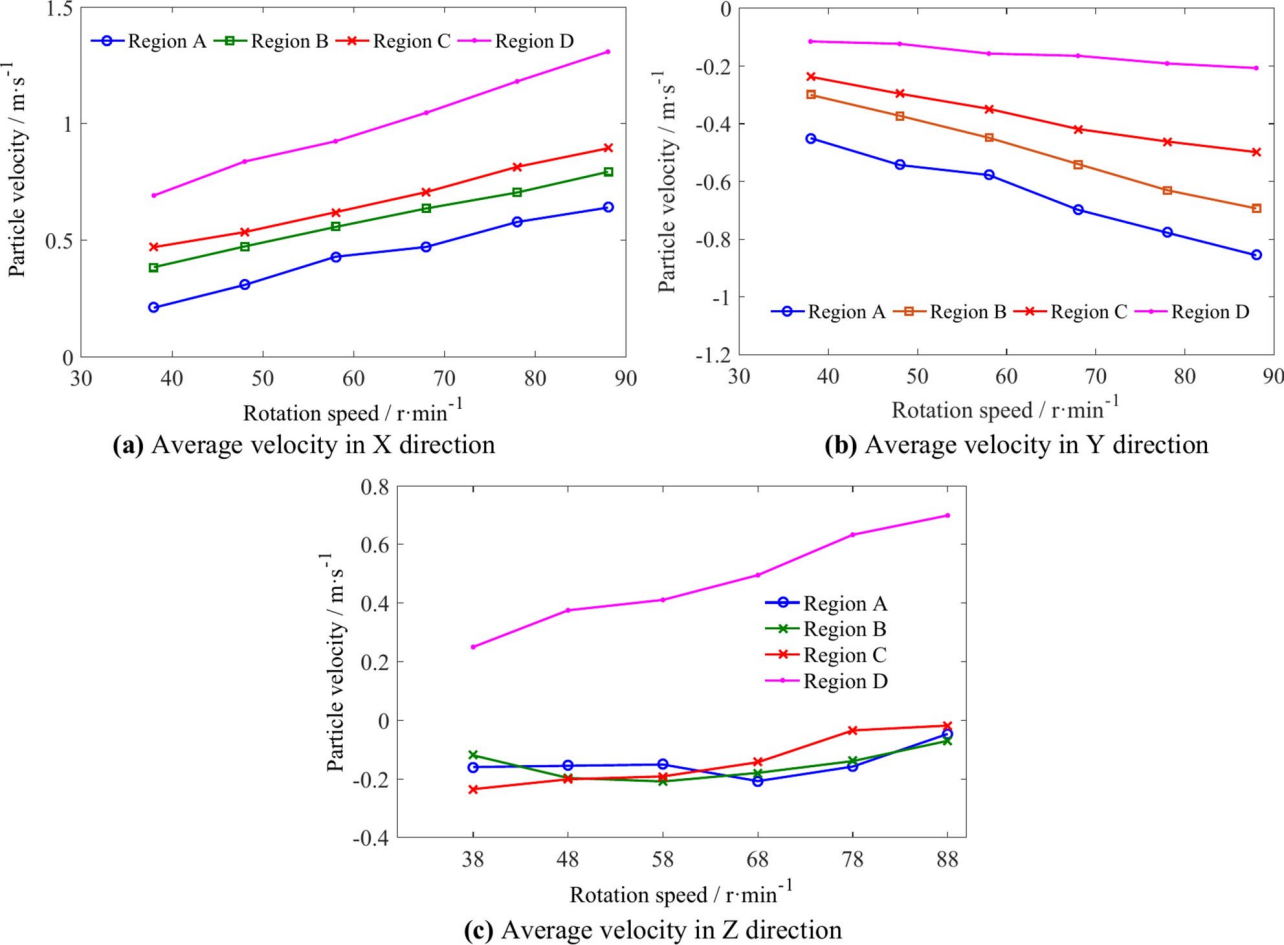
The influence of rotation speed on average velocity of coal particles in regions A, B, C and D.

The average velocities of coal particles in regions A, B, C and D is shown in Table 9. With the increasing of the rotational speed, the velocities of coal particles in X direction and Y direction in the four regions increase continuously, and the velocity in Z direction in region D increases obviously. The velocity in the Z direction of the coal particles in regions A and B first increases and then decreases, while that in region C continues to decrease, as shown in Fig. 26.

**Table 9:** The average velocities of coal particles in regions A, B, C and D.

Rotation speed/r min^−1^		Average velocity/m s^−1^			
		Region A	Region B	Region C	Region D
4	X	0.211	0.385	0.471	0.692
	Y	− 0.450	− 0.300	− 0.237	− 0.115
	Z	− 0.160	− 0.120	− 0.235	0.250
5	X	0.309	0.474	0.535	0.838
	Y	− 0.543	− 0.372	− 0.296	− 0.123
	Z	− 0.155	− 0.197	− 0.201	0.375
6	X	0.429	0.557	0.620	0.925
	Y	− 0.578	− 0.449	− 0.348	− 0.157
	Z	− 0.151	− 0.209	− 0.191	0.411
7	X	0.472	0.637	0.707	1.047
	Y	− 0.698	− 0.540	− 0.418	− 0.165
	Z	− 0.208	− 0.179	− 0.143	0.495
8	X	0.578	0.705	0.815	1.181
	Y	− 0.778	− 0.630	− 0.462	− 0.191
	Z	− 0.158	− 0.139	− 0.034	0.633
9	X	0.640	0.793	0.895	1.309
	Y	− 0.856	− 0.693	− 0.498	− 0.207
	Z	− 0.046	− 0.070	− 0.018	0.699

### The influence of motion parameters on coal loading rate

**Fig. 27:**
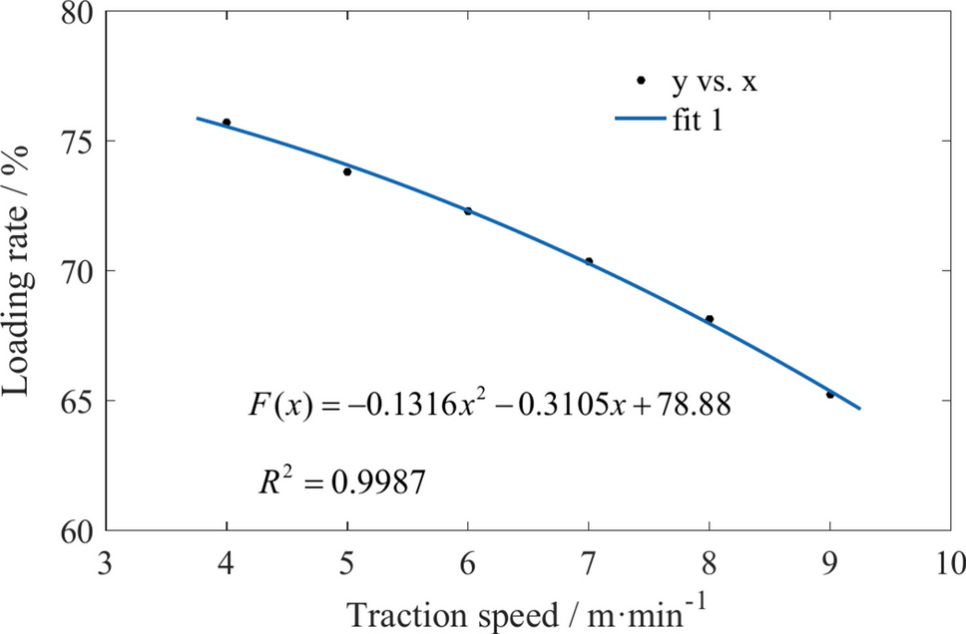
The variation of coal loading rate with different traction speeds.

**Fig. 28:**
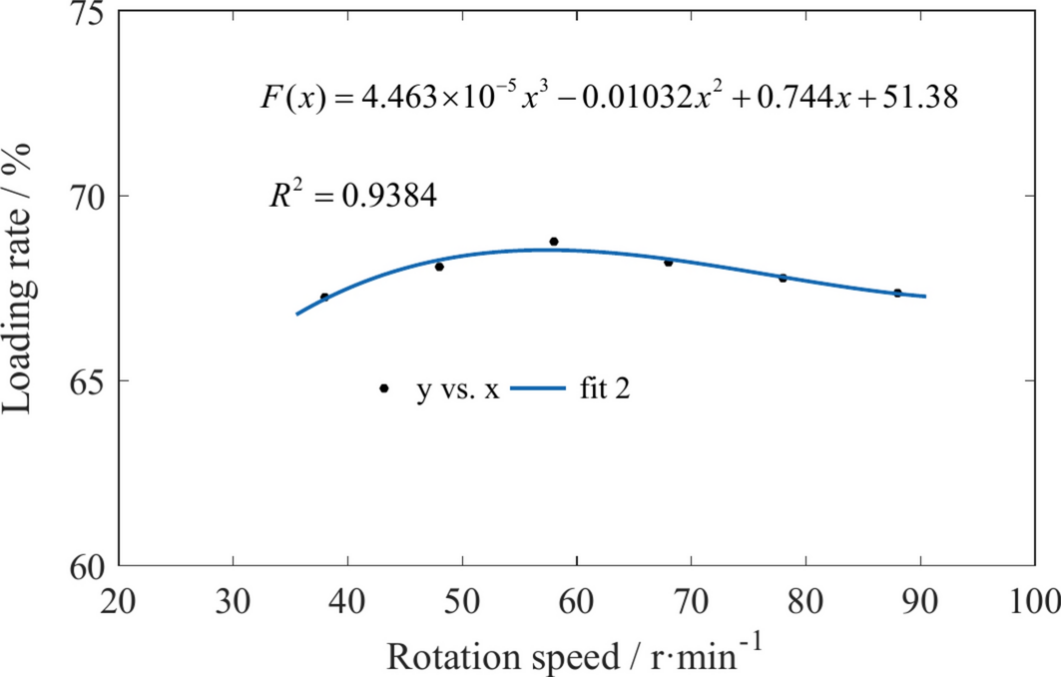
The variation of coal loading rate with different rotation speeds.

By statistically analyzing the coal particle mass in regions 1 and 2 of Fig. 2, the effects of traction speed and drum rotation speed on coal loading rate were obtained, as shown in Figs. 27 and 28. Due to the increase in traction speed, the number of coal particles in the envelope region continues to increase per unit time. The velocity of coal particles moving outward along the drum axis in each region does not increase, resulting in a continuous decrease in the coal loading rate as the traction speed increases. An increase in the rotation speed can improve the ability of coal particles to flow to the scraper conveyor, but the velocity of coal particles in other directions also increases. The coal loading rate first increases and then decreases with increasing rotation speed.

**Fig. 29:**
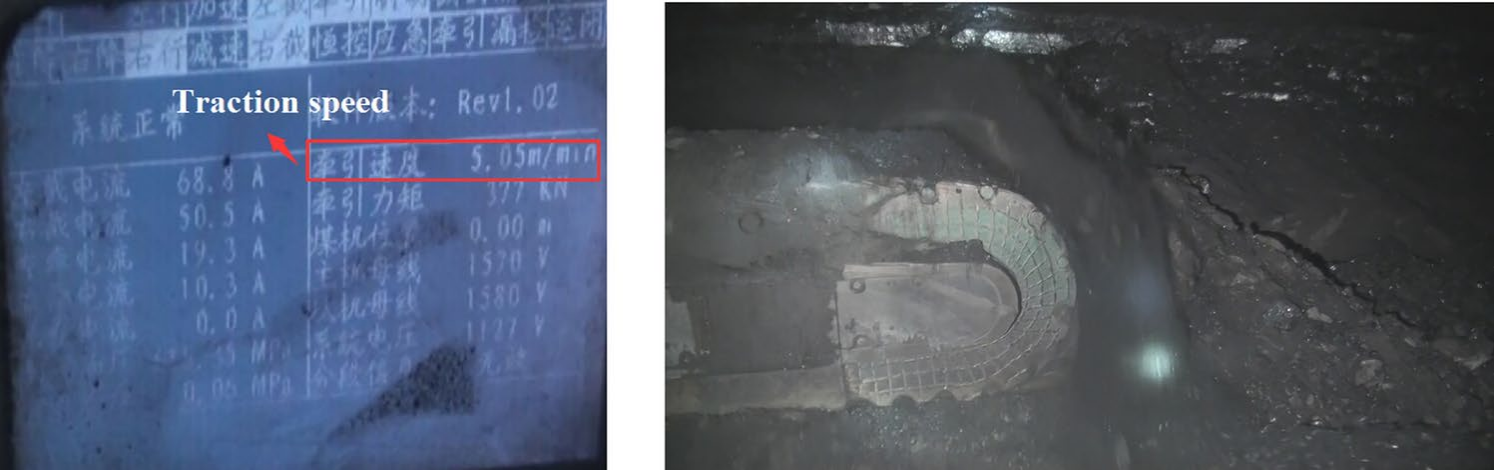
Underground cutting test.

Based on the above analysis results, an industrial underground test was conducted at a traction speed of 5m/min and a drum rotation speed of 58 r/min, as shown in Fig. 29. The statistics of the coal falling amount and the coal transportation amount of the scraper conveyor in a single cutting face length of the shearer showed that the coal loading rate of the spiral drum was 68.75%. Through discrete element numerical simulation, it was found that the coal loading rate of the drum during the cutting process was 72.1%. The error between the experimental and simulated coal loading rates is 4.51%. The main reason for the error is that the numerical simulation did not consider the incomplete transport of particles between the scraper conveyor and the coal wall, resulting in an overestimation of the drum coal loading rate calculation.

## Conclusion

Based on the measurement results of the coal sample properties, the intrinsic parameters of the material were calibrated, and construct a three-dimensional bonding contact model of coal wall and a discrete element model of spiral drum coal loading were constructed. Through numerical simulation, the movement state of coal particles in the loading process was obtained. The coal loading rate of the spiral drum was determined through statistical analysis of coal particle mass distribution across various regions. The numerical simulation results were validated through industrial underground experiments.

Spatial segmentation of the spiral drum’s envelope region along radial and axial directions was conducted to characterize coal particle motion during loading. Through statistical analysis of the number and velocity of coal particles in different regions, it was found that there are more coal particles in the upper space near the coal wall in the envelop region of the spiral drum. Increasing the velocity of coal particles along the axial direction of the drum in this region can improve the coal loading rate. In order to further analyze the movement of coal particles in the area, the region was divided into four small regions again. The coal particles near the end plate had the lowest velocity along the axial direction of the drum, and the coal particles in this region were mostly thrown towards the goaf. Increasing the traction speed will lead to an increase in coal particles entering the envelope area of the spiral drum per unit time, while reducing the speed of coal particles along the axial direction of the drum. The increase in the rotation speed of the spiral drum can improve the ability of coal particles to flow out of the spiral drum along the axial direction, thereby improving coal loading efficiency.

According to the research results, the coal shearer can achieve a reasonable combination of traction speed and rotation speed to achieve better coal loading effect. In the following research, we will analyze the influence of different spiral blade angles and hub diameters on the coal particle motion characteristics, and seek the optimal structure and motion parameters to improve the coal loading efficiency of the spiral drum.

## Data Availability

The datasets used and analysed during this study are available from the corresponding author on reasonable request.
